# Secondary structure assignment of proteins in the absence of sequence information

**DOI:** 10.1093/bioadv/vbab038

**Published:** 2021-11-29

**Authors:** Sammy Khalife, Thérèse Malliavin, Leo Liberti

**Affiliations:** LIX, CNRS, Ecole Polytechnique, Institut Polytechnique de Paris, Palaiseau 91128, France; CNRS, Institut Pasteur UMR 3528, Paris 75015, France; LIX, CNRS, Ecole Polytechnique, Institut Polytechnique de Paris, Palaiseau 91128, France

## Abstract

**Motivation:**

The structure of proteins is organized in a hierarchy among which the secondary structure elements, *α*-helix, *β*-strand and loop, are the basic bricks. The determination of secondary structure elements usually requires the knowledge of the whole structure. Nevertheless, in numerous experimental circumstances, the protein structure is partially known. The detection of secondary structures from these partial structures is hampered by the lack of information about connecting residues along the primary sequence.

**Results:**

We introduce a new methodology to estimate the secondary structure elements from the values of local distances and angles between the protein atoms. Our method uses a message passing neural network, named Sequoia, which allows the automatic prediction of secondary structure elements from the values of local distances and angles between the protein atoms. This neural network takes as input the topology of the given protein graph, where the vertices are protein residues, and the edges are weighted by values of distances and pseudo-dihedral angles generalizing the backbone angles ϕ and *ψ*. Any pair of residues, independently of its covalent bonds along the primary sequence of the protein, is tagged with this distance and angle information. Sequoia permits the automatic detection of the secondary structure elements, with an *F*1-score larger than 80% for most of the cases, when *α* helices and *β* strands are predicted. In contrast to the approaches classically used in structural biology, such as DSSP, Sequoia is able to capture the variations of geometry at the interface of adjacent secondary structure element. Due to its general modeling frame, Sequoia is able to handle graphs containing only $C_{\alpha }$ atoms, which is particularly useful on low resolution structural input and in the frame of electron microscopy development.

**Availability and implementation:**

Sequoia source code can be found at https://github.com/Khalife/Sequoia with additional documentation.

**Supplementary information:**

Supplementary data are available at *Bioinformatics Advances* online.

## 1 Introduction

Since three decades, the development of structural biology has been driven by the intention to relate the function of molecular objects to the physico-chemical rules at the atomic level. In that frame, tools for the geometric analysis of the protein graph, consisting of atoms and residues, are essential. The protein structure is historically described as a hierarchy of molecular objects: (i) the individual protein residue; (ii) the secondary structure elements (*α* helices, *β* strands and loops), which are formed by stretches of residues covalently connected according to the sequence order; (iii) the combination of secondary structure elements, such as the parallel or anti-parallel *β* sheets formed from associations of *β* strands through hydrogen bonds; (iv) the tertiary structural motifs (Andreeva *et al.*, 2020; El-Gebali *et al.*, 2019; Sillitoe *et al.*, 2021), where the association of secondary structure elements is most often stabilized through the formation of a hydrophobic core between residue sidechains; and (v) the quaternary structure, where protein domains and/or individual proteins or biomolecules interact to form larger molecular assemblies. The levels (iv) and (v) of the hierarchy define the 3D structure of folded proteins or of assemblies of folded proteins. It should be noted that this hierarchy is strongly based on a description of proteins as polymers, formed of a succession of covalently bonded amino acids. The sequence information coupled to the secondary structure elements was also used for modeling the order-to-disorder transition (Dan *et al.*, 2010). Moreover, the succession of protein residues along the primary sequence is often used as an input to classical methods for secondary structure prediction (Frishman and Argos, 1995; Kabsch and Sander, 1983), in particular to detect hydrogen bonds between backbone atoms, and to characterize the *α* helices and *β* strands. To the best of our knowledge, all of the current methods for the determination of secondary structure from inter-atomic distances and angles also use the amino acid sequence assignment. In this work, we propose to bypass the sequence information.

Our work is motivated by the fact that within the primary sequence of a protein, parts are often missing in the structure. For example, disordered regions of proteins are not visible in electronic density maps obtained using X-ray crystallography or electronic microscopy (EM). Another aspect of missing information is encountered in low resolution structures obtained by X-ray crystallography or EM where only a partial number of protein atoms is present, such as, e.g. the *α* carbons.

During the last decade, the explosion of the fields of artificial intelligence and machine learning has driven the consistent development of methods coming from these fields and applied to biology problems. Graph representations combined with deep-learning methods or generative models have proved to be relevant for several applications dealing with the complex geometry of protein structures, such as protein–ligand interaction (Lim *et al.*, 2019) or protein design (Ingraham *et al.*, 2021). In order to harness their experimental performance, we propose a message passing approach to integrate geometric features of proteins into a convolutional graph neural network (GNN), which automatically detects the type of secondary structure elements (*α* helices, *β* strands and loops) using the distance and angle information between heavy backbone atoms as its sole input. Specifically, we do not consider any input coming from the existence of covalent bonds between successive residues along the primary sequence. Consequently, the approach can be applied to structures that are determined only partially. We also point out that this is a methodological rather than biological study. Consequently, we aim at showing that our proposed methodology works well in general, meaning we do not fine-tune it for specific proteins.

The approach proposed here, named *Sequoia*, is computationally tested on protein structures determined using X-ray crystallography or nuclear magnetic resonance (NMR). We evaluate the effect of noise level in the input data, as well as the prediction efficiency of Sequoia for various secondary structure elements and protein graphs. On all-atom protein structures, Sequoia predicts *α* helices and *β* strands with *F*1 scores respectively mostly better than 95% and 90% and the joint prediction of *α* helices and *β* strands displays a *F*1 score mostly larger than 80%. One should notice that this comparison is calculated with respect to the results with DSSP (Frishman and Argos, 1995; Kabsch and Sander, 1983). Sequoia also displays robustness with respect to noisy inputs and missing residues in the graph, as well as for sparse $C_{\alpha }$ graphs. Interestingly, most of our prediction errors are observed for residues located at the extremities of secondary structure elements. Indeed, these residues undergo continuous geometrical transformations, which makes them difficult to predict in the classical discontinuous description from Frishman and Argos (1995) and Kabsch and Sander (1983).

The rest of this article is organized as follows. Section 2 presents the protein descriptors, their robustness to noisy measurements and the Sequoia architecture, along with a simple but solid baseline named first order statistics (FOS). Section 3 describes the results. Discussions and conclusions are given in Section 4.

## 2 Methodology

### 2.1 Graph description

We consider a natural geometric representation of molecules with *n* atoms in terms of an n×3*realization matrix* where the *i*-th row is a vector in ${\mathbb{R}}^{3}$ corresponding to the Euclidean position of the *i*-th atom of the molecule, for i≤n. This representation corresponds to the steady state of the protein, enforcing a *molecular rigidity assumption* (Luisi, 1977). For the Sequoia prediction purposes, we represent such structure by means of a simple, undirected, edge-weighted graph G=(V,E,d), where *V* is the set of atoms, and *E* is the set of atom pairs {*i*, *j*} with known distance value *d_ij_*. A graph is a very relevant model for describing protein structure and has been widely used (Di Paola and Giuliani, 2015; Fout *et al.*, 2017; Heal *et al.*, 2018; Huan *et al.*, 2004; Krishnan *et al.*, 2008; Mason and Verwoerd, 2007).

Two different networks will be considered: one full network with all heavy backbone atoms and one simplified network containing only the Carbons *α*. In the full network, the heavy atoms are grouped into to subsets corresponding to protein residues, in a way similar to the definition of spin systems in NMR (Lian and Roberts, 2011).

The graph of residues will be defined by two methods:

-
A : a *k*-nearest neighbors graph $G_{k}=(V,A)$, where *V* is the set of all residues in the protein and $(r_{1},r_{2})\in A$ if and only if *r*_2_ is one of the *k*-nearest neighbors of *r*_1_;
-
B : a threshold based graph $G\prime_{\tau }=(V,E)$, where *V* is the set of all residues in the protein and $\{r_{1},r_{2}\}\in E$ if and only if the measured distance between *r*_1_ and *r*_2_ is lower than the threshold *τ*.

Both of these constructions require the notion of distance between two residues. In the following, we define the distance between two residues as *the minimum distance between the respective atoms composing them*.

Note that the method B is formally undirected—which may not be the case for A, and the threshold *τ* can be set to a value corresponding to the physical requirements of structural biology. Our experiments revealed that both methods lead to very similar results when *k *=* *2 in A and *τ*  =  3 Å in B, and decided to use method A to build the graph of the protein structure.

In addition to distance information, angle information between heavy backbone atoms will be added to the edges of the protein graph. The selected angles will be a generalization of the backbone dihedral angles ϕ and *ψ*, described below. This generalization will permit the computation of these angles for any pairs of protein residues, covalently bonded or not. In that way, no information on primary sequence connectivity of the protein is present in the graph input of the neural network.

### 2.2 Protein descriptors and neural network inputs

The backbone dihedral angles ϕ and *ψ* are classically defined between the atoms belonging to successive residues *r* *−* 1, *r* and *r *+* *1 in the protein primary sequence:

- The carbon atom of the carbonyl group from residue *r* *−* 1, the nitrogen atom, the carbon-*α* atom and the carbon atom of the carbonyl group from residue *r.*
- The nitrogen atom, the carbon-*α* atom and the carbon atom of the carbonyl group from residue *r* and the nitrogen atom from residue *r* + 1.

In this work, this definition will be generalized to any couple of residues being closer in the space than the threshold *τ*.

Using the atomic coordinates determining the protein structures, it is straightforward to determine the dihedral angles. However, in the case when only the distances between atoms are known, it can be shown that using poly-spherical coordinates (Porogelov, 1987), or alternatively a Clifford algebraic formulation (Lavor *et al.*, 2015), the cosine of the dihedral angles cos ⁡ω can be computed using only distances between atoms.

If *ω* represents the dihedral angle between two planes defined by four atoms {i−3,i−2,i−1,i}, where the first plane is defined by *i* −3, *i* −2, *i* −1, and the second by *i* −2, *i* −1, *i*, the cosine law for trihedron [Supplementary Fig. S1 and Lavor *et al.* (2015)] can be written in the following way:

(1) cos ⁡γ=cos ⁡α cos ⁡β+sin ⁡α sin ⁡β cos ⁡ω,

where *α*, *β* and *γ* are angles between vectors made by the pairs of atoms in the following sense. If *x_k_* is the 3D-positional vector of atom *k*, then *α* is the angle between vectors $\left(x_{i-3}-x_{i-2}\right)$ and $\left(x_{i-1}-x_{i-2}\right)$. *β* is the angle between $\left(x_{i-1}-xi-2\right)$ and $\left(x_{i}-x_{i-2}\right)$, and *γ* the angle between $\left(x_{i-3}-x_{i-2}\right)$ and $\left(x_{i}-x_{i-2}\right)$. This is displayed in Supplementary Figure S1.

Using relation between cos ⁡ and sin ⁡:

(2) $$\text{cos}\;\omega =\frac{\;\text{cos}\;\gamma -\text{cos}\;\alpha \;\text{cos}\;\beta }{\sqrt{1-{\text{cos}}^{2}\;\;\alpha }\sqrt{1-{\text{cos}}^{2}\;\;\beta }}.$$

Furthermore, using the planar cosine law, cos ⁡α,  cos ⁡β and cos ⁡γ are given by:

(3) $$\text{cos}\;\left[\begin{array}{c} \alpha \\ \beta \\ \gamma \end{array}\right]=\left[\begin{array}{c} f(d_{i-1,i-2},d_{i-2,i-3},d_{i-3,i-1})\\ f(d_{i-1,i-2},d_{i-2,i},d_{i-1,i})\\ f(d_{i-3,i-2},d_{i-2,i},d_{i-3,i}) \end{array}\right],$$

where $d_{i,j}$ is the distance between atoms *i* and *j*, and:

(4) $$f(x,y,z)=\frac{-z^{2}+x^{2}+y^{2}}{2xy}.$$

Using Equations (3) and (4), Equation (2) can be reformulated as:

(5) $$\text{cos}\;\omega =\frac{2d_{i-2,i-1}^{2}\Delta_{i}-(d_{i-3,i-2,i-1})(d_{i-2,i-1,i})}{\Gamma_{i}\sqrt{4d_{i-2,i-1}^{2}d_{i-2,i-3}^{2}-(d_{i-2,i-1,i}^{2})}}.$$

with:

$$\begin{array}{c} d_{i-3,i-2,i-1}=d_{i-3,i-2}^{2}+d_{i-2,i-1}^{2}-d_{i-3,i-1}^{2}\\ d_{i-2,i-1,i}=d_{i-2,i-1}^{2}+d_{i-2,i}^{2}-d_{i-1,i}^{2}\\ \Delta_{i}=d_{i-3,i-2}^{2}+d_{i-2,i}^{2}-d_{i-3,i}^{2}\\ \Gamma_{i}=\sqrt{4d_{i-1,i-2}^{2}d_{i-2,i}^{2}-(d_{i-3,i-2,i-1}^{2})}. \end{array}$$

Equation (5) allows the calculation of backbone angles ϕ and *ψ* depending on the set of considered atoms i−3,i−2,i−1 and i−2,i−1,i, as recalled at the beginning of the subsection. Thus, using this Equation, we generalize the notion of ϕ and *ψ* angles to any pair of residues *k* and *l* in the protein, by considering the relevant atoms in the residues. Then if residues *k* and *l* are connected in the graph, the edge features *x_kl_* are defined as $x_{kl}=(d_{kl},\;\text{cos}\;\phi_{kl},\;\text{cos}\;\psi_{kl})$ from the distance *d_kl_* between the two residues, and the cosines of the pseudo-dihedral angles $\phi_{kl}$ and *ψ_kl_*. The equations described above are used in the definition of the interval Branch-and-Prune (iBP) algorithm for listing protein conformations consistent with distance data (Lavor *et al.*, 2012; Liberti *et al.*, 2014).

In addition to a graph containing all backbone atoms, we also tested the prediction of secondary structure on a simplified graph containing only $C_{\alpha }$ atoms. In that case, the edge between $C_{\alpha }$ atoms of residues *a* and *b* is labeled by $x_{ab}=(d_{ab},\;\text{cos}\;\Phi_{ab})$, *d_ab_* being the distance between $C_{\alpha }$ atoms and one pseudo-dihedral angle $\Phi_{ab}$ being defined using the Equation (5) where atoms *i* −1 and *i* −2 are the atoms $C_{\alpha }$ of residues *a* and *b*, the atoms *i* and *i* −3 being two different atoms $C_{\alpha }$ the closest respectively of atoms *i* −1 and *i* −2.

### 2.3 Testing the noise robustness of dihedral angle computation

In practice, imprecision on distance measurements may lead to greater errors in the dihedral angle estimates. Indeed, the imprecision will lead to numerical errors on cos ⁡ω as the cosine law for trihedron [Equation (1)] is no more valid. The relationship between the inter-atomic distances and the dihedral angle *ω* [Equation (5)] can be reformulated as a functional relationship: cos ⁡ω=g(D), where *D* is the matrix containing all distances between the atoms *i*, *i* −1, *i* −2 and *i* −3. An estimation of noisy dihedral angles can be obtained with the following equation:

$${\left(\text{cos}\;\omega \right)}_{\epsilon }=g(\mathrm{proj}(D+\epsilon )),$$

where *ϵ* is a (4,4) symmetric matrix verifying: $\forall i,\epsilon_{i,i}=0,\;\forall i<j,\epsilon_{i,j}∼N(0,\eta_{1})$, and proj is the projection operator onto the cone of symmetric positive semidefinite (PSD) matrices. The proj operator avoids to consider matrices representing non-Euclidean 3D objects, in which case, the denominator of the right hand side in Equation (5) could be zero.

Algorithm 1 proj operator onto the cone of symmetric PSD matrices

1: **Input** *D*: Symmetric matrix

2: **Output**$D_{\mathrm{proj}}$: Projected matrix onto the cone of symmetric PSD matrices

3: $G=-\frac{1}{2}JDJ$, where $J=I_{n}-\frac{1}{n}11^{⊤}$ (*n*: number of atoms), with $G=PDP^{-1}$, *P* real matrix such that $PP^{-1}=I_{n}$

4: $D_{+}$ diagonal matrix such that $D_{+}(i,i)=\max(0,D_{i,i})$

5: $E=PD_{+}P^{-1}$

6: $D_{\mathrm{proj}}=1\mathrm{diag}{\left(E\right)}^{T}-2E+\mathrm{diag}(E)1^{⊤}$

7: Return $D_{\mathrm{proj}}$

To transform the matrix *D* to a matrix corresponding to a Euclidean 3D molecular object, the proj operator takes as input a symmetric matrix *D*, and returns its projection $D_{\mathrm{proj}}$ onto the cone of symmetric PSD matrices. This projection is obtained using the procedure described in Algorithm 1 [see Dokmanic *et al.* (2015) for details about this transformation].

In order to estimate the impact of noise addition, we conducted experiments on the ϕ angles of the first 25 residues of a protein [Protein Data Bank (PDB) entry: 1M22] extracted from Dataset A (presented in Section 2.8). Results obtained for a thousand Monte-Carlo simulations are depicted in Supplementary Figure S2A for noise levels $\eta_{1}\in \{0.05,0.1\}$ Å. We conclude that noisy distances will impact significantly dihedral angles when the imprecision is >0.05 Å.

Equation (2), formulated as cos ⁡ω=h(α,β,γ), shows that the dihedral angle *ω* can be computed only based on angles *α*, *β* and *γ*. If these angles were to be computed with another method than distances, the impact on the dihedral angles might be reduced. In order to evaluate the robustness of our features to the imprecision on angles *α*, *β* and *γ* (Supplementary Fig. S1), we conducted a similar experimental analysis:

cos ⁡ω=h((α,β,γ)+ϵ),

where $\epsilon \in {\mathbb{R}}^{3}∼N(0,\eta_{2}\times 1)$, with N being the normal distribution, 0=(0,0,0), 1=(1,1,1), and *η*_2_ being the relative amplitude of the noise on the cosines. Similarly to the evaluation of noise effect on distances, we considered a thousand Monte-Carlo simulations. The results are depicted in Supplementary Figure S2B for noise levels $\eta_{2}\in \{0.05,0.1\}$. They show that adding noise to angles *α*, *β* and *γ* has less impact to dihedral angles *ω* than adding noise to the distances between atoms *i*, *i* *−* 1, *i* *−* 2 and *i* *−* 3. Following the results of these numerical experiments, the robustness to noise of Sequoia will be tested in the following by adding noise to cos ⁡ω. The error induced on *ω* by adding noise on cos ⁡ω was also estimated using Monte-Carlo simulations (Supplementary Fig. S2C). Depending on the regions of *ω* values and on the noise level *η*_2_, the error was comprised between 3 and 14°.

### 2.4 Simple baseline with FOS

A FOS, considered as the baseline for the prediction of secondary structure, was defined for comparison purposes with Sequoia. For a fair comparison (Section 2.5), the baseline will also be sequence agnostic. FOS considers the neighborhood of a residue in the graph and computes the average and variances of the cosine of the dihedral angles ϕ and *ψ* in this neighborhood. The average and variances are then used as features for supervised classification as further explained in Section 2.6.

The idea of this baseline is based on the following remark. Along each *β* strand element, the protein backbone extends locally in a straight direction whereas along *α* helices, the backbone displays locally a spiral. These very different local geometries should have an impact on the moving average of cosine of dihedral angles ϕ and *ψ*, which leads to the FOS definition.

### 2.5 Sequoia: a message passing neural network

One of the advantages of modeling the protein as a graph of residues is to harness the experimental performance of GNNs. For the sake of generality, we adopt the formulation of message passing (Gilmer *et al.*, 2017), which describes the core idea of GNNs. In the following, the variable *t* represents a time increment of the parameters of the model, and $h_{v}^{t}$ the hidden variable state of node *v* at time *t*. The initial hidden states of the model $h_{v}^{0}$ are set to the features considered, which in the frame of this article are the cosines of the pseudo-dihedral angles between residues. During the message passing phase, the hidden states $h_{v}^{t}$ of each node in the graph are updated based on messages $m_{v}^{t}$ according to

(6) $$m_{v}^{t+1}=\sum_{w\in N(v)}M_{t}(h_{v}^{t},h_{w}^{t},e_{v}^{w})h_{v}^{t+1}=U_{t}(h_{v}^{t},m_{v}^{t+1}),$$

where *M_t_* is a message function and *U_t_* a vertex update function. After *T* iterations, the final output of the node is computed with a readout function *R*:

$$y_{v}=R(h_{v\in G}^{T}).$$

The choice of the family of *M_t_* and *U_t_* and *R* lead to the design of the GNN, as explored in several references for various applications [e.g. Convolutional Network (Duvenaud *et al.*, 2015), Gated Graph Neural Network (Li *et al.*, 2016) or Molecular Graph Convolutions (Kearnes *et al.*, 2016)]. The learning of the parameters is then performed using standard back-propagation, interpreting the parameter *t* as the index of the neural network layer. The choice of functions *M_t_*, *U_t_* and *R* for our experiments is described in Section 2.7.2.

### 2.6 Secondary structure prediction with node classification

Based on our formulation, the attribution of a secondary structure to a residue can naturally be formulated as a node classification problem. If *y* represents the label variable, then we consider three situations:

- *α*-None: attribution to an *α* helix element. y∈{0,1}.
- *β*-None: attribution to a *β* strand element. y∈{0,1}.
-
α−β− None: attribution to an *α* helix, to a *β*-strand or to other. y∈{0,1,2}.
- All: attribution to all secondary structure elements defined in DSSP (Kabsch and Sander, 1983), leading to eight classes: y∈{0,…,7}.

On the one hand, the FOS method translates into a simple classification problem that we approach with standard supervised learning methods. On the other hand, the message passing neural network (MPN) method leads to the training of a MPN. The details of the classifier used for FOS and MPN architecture are described below. The training on the Datasets A and B has been organized in the following way: 70% of randomly chosen proteins from Dataset A were used for training and the remaining part for testing. The proteins from Dataset B were only used for testing.

### 2.7 Practical implementation

#### 2.7.1 First order statistics

As detailed in Section 2.4, the FOS formulation leads to a simple classification problem with features belonging to ${\mathbb{R}}^{3}$. We used a *k*-nearest neighbor as the classifier for our baseline.

#### 2.7.2 Message passing neural network

The design of our MPN is based on the continuous kernel-based convolutional operator from Gilmer *et al.* (2017), also known as the edge-conditioned convolution from Simonovsky and Komodakis (2017). Our implementation is based on the two high-level APIs pytorch (Paszke *et al.*, 2017) and pytorch-geometric (Fey and Lenssen, 2019).

We used a two layer kernel-based convolutional, where two message passing schemes are performed sequentially on the hidden states. In our case, for each of the two layers, the message function *M_t_* and the vertex update function *U_t_* are defined as:

$$\begin{array}{c} M_{t}(h_{v}^{t},h_{w}^{t},e_{v}^{w})=h_{w}^{t}\;.\;N(e_{v}^{w})\\ U_{t}(h_{v}^{t},m_{v}^{t+1})=\Theta \;.\;h_{v}^{t}+m_{v}^{t+1}, \end{array}$$

where N is a four-layer linear perceptron with Rectifier Linear Unit activations between each layer and Θ is a linear operator. Finally, the readout function *R* is a softmax function composed with a two-layer linear perceptron to output after the two main layers a predicted label *y_v_* for each *v*.

Our initial formulation leads to 1D discrete node feature corresponding to the type of amino acid residue for the node and the edge features defined above as *x_kl_* and *x_ab_* and containing distances and cosines of pseudo-dihedral angles. However, we noticed a gain in performance by aggregating edge features in the neighborhood of a node into its features. This behavior is somehow similar to the experiments led in Gilmer *et al.* (2017), where edge features constructed from the node features were added to the graph. In our case, the transformation goes from edges to nodes. We conjecture it to be a consequence of data augmentation (Chen *et al.*, 2020).

### 2.8 Datasets of protein structures

#### 2.8.1 Dataset A

Dataset A is composed of 3621 protein X-ray crystallographic structures downloaded from the server PISCES (Wang and Dunbrack, 2003). These structures correspond to a set of PDB (Berman *et al.*, 2000) entries for which structures have been determined at a resolution better than 1.6 Å, and with R factors better than 0.25. The set of PDB entries and protein chains present in Dataset A has been chosen (Wang and Dunbrack, 2003) in order that the percentage of sequence identity between any pair of chains is smaller than 20%, to avoid statistical bias on the protein sequences.

#### 2.8.2 Dataset B

Dataset B is composed of 226 protein structures obtained by processing the database of NMR chemical shifts used for the training of the neural network TALOS-N (Shen and Bax, 2015). For 226 proteins of this database, a structure was determined by NMR. We decided to pick up the first conformer of these NMR structures to build a NMR structure database. The list of proteins and chains used in Datasets A and B is available in the Supplementary Material.

### 2.9 Validation of sequoia results

The secondary structures predictions obtained using Sequoia were compared to the output of DSSP (Kabsch and Sander, 1983), a classical software for the determination of secondary structures. Training samples corresponds to 70% of the samples in Dataset A, and Test A corresponds to the 30% remaining samples. Test B corresponds to the whole Dataset B.

#### 2.9.1 Evaluation metrics

To evaluate the performance of Sequoia and compare it to our baseline, we use the *F*1-score, which is the geometric mean between recall and precision. Recall and precision extend to the multi-class case, and so does *F*1. In a problem with *d* classes, let *P_i_* be the ratio of samples correctly assigned to the class *i* over the number of samples assigned to the class *i*. Let *R_i_* be the ratio of samples correctly assigned to the class *i* over the true number of samples within class *i*. Then recall, precision and *F*1-score are defined as

$$R=\frac{1}{d}\sum_{i=1}^{n}R_{i}\;\;P=\frac{1}{d}\sum_{i=1}^{d}P_{i}\;\;F1=\frac{2(R\times P)}{\left(R+P\right)}.$$

#### 2.9.2 Use of sequoia on information coming from EM maps

Predictions were also realized in the context of low resolution structural information, by analyzing positions of atoms $C_{\alpha }$ predicted from EM maps. To do so, we used the output of a deep-learning approach, Deeptracer (Pfab *et al.*, 2021; Si *et al.*, 2020), which predict positions of protein atoms from the image recorded from EM single particle analysis. Several entries from the Electronic Microscopy Data Bank (EMDB), which will be described below, were used as inputs for the Web server of Deeptracer (https://deeptracer.uw.edu/home), and the early output containing only atoms $C_{\alpha }$ was used to feed Sequoia.

## 3 Results

The results obtained by Sequoia will be compared to a FOS, defined as the average and variances of the cosine of the dihedral angles ϕ and *ψ* in the neighborhood of a residue. The predictions are run on two datasets of protein structures: the Dataset A composed of 3621 protein X-ray crystallographic structures downloaded from the server PISCES (Wang and Dunbrack, 2003) and the Dataset B composed of 226 protein structures obtained by processing the database of NMR chemical shifts used for the training of the neural network TALOS-N (Shen and Bax, 2015). Several classifications of secondary structure elements have been predicted: *α*-Other assigning *α* helix elements, *β*-Other assigning *β* strand elements, *α*-*β*-Other assigning *α* helix and *β* strand elements and All assigning all secondary structure elements defined in DSSP (Kabsch and Sander, 1983).

### 3.1 Prediction of secondary structure elements

**Fig. 1.**
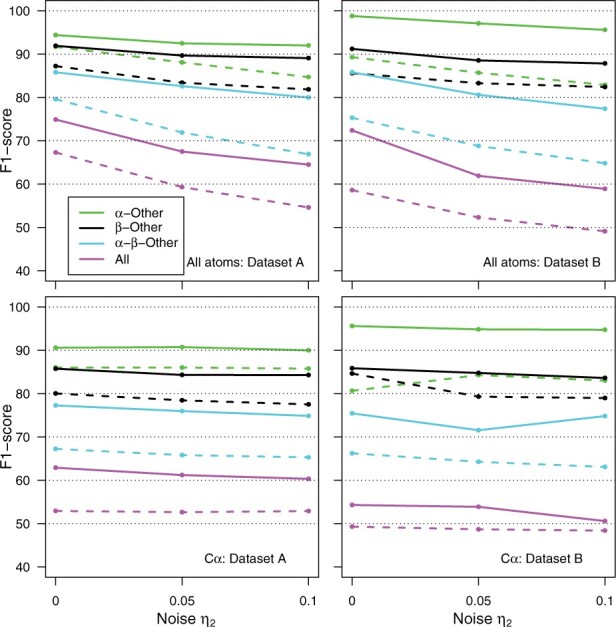
Sequoia and FOS predictions of secondary structure elements. The predictions (*F*1-score) are displayed for all atoms (upper panels) and atoms $C_{\alpha }$ (lower panels), for Datasets A (left panels) and B (right panels) and for Sequoia (continuous lines) and FOS (dashed lines). The Datasets A and B are described in details at the end of Supplementary Material. The *F*1-scores are plotted according to the noise level added to cos ⁡ω (Section 2.3)

Several experiments have been conducted to investigate the efficiency of Sequoia. First, the Sequoia results have been compared to the FOS baseline in order to estimate the performance improvement brought by a cutting-edge machine learning approach (Fig. 1).

The results obtained for secondary structure assignment are reported in Figure 1 for the noise-free tests, and for the noise level $\eta_{2}\in \{0.05,\;0.1\}$. The FOS method, introduced in Section 2.4, provides a solid baseline with prediction success rates (dashed lines) larger than 50% for graphs with all backbone atoms, in three cases: *α*-Other, *β*-Other and *α*-*β*-Other. In the case of noise-free test the best *F*1-scores are obtained using *k *=* *20 in the nearest neighbors classifier, whereas for the test in presence of noise, *k *=* *60 is required in the classifier to obtain the best *F*1-scores.

Interestingly, the MPN Sequoia (Fig. 1, continuous lines) provides improvement with respect to FOS by a wide margin (5% to more than 10%). The best improvement is obtained for the classification *α*-*β*-Other (cyan curves). Furthermore, the improvement increases with the addition of noise, which proves Sequoia is more robust.

The best prediction results are obtained in all cases for the classification *α*-Other (Fig. 1: green continuous curves). This is certainly due to the very narrow interval of dihedral angles corresponding to the definition of the *α*-helix, which makes the angles values more discriminant. Adding the *β*-strand (black and cyan curves) induces a decrease of success rate as the dihedral angles defining the *β*-strand sample larger value intervals. Finally predicting a full classification requires to take into account the whole set of dihedral values measured in the proteins, which sample much larger intervals and display large heterogeneity in regions outside of *α*-helices and *β*-strands. Consequently, the results obtained for predicting the eight types of secondary structure described in DSSP (magenta curves) are, in all cases, behind from other predictions by 10–20%. This behavior is expected as the power of a classification approach heavily depends on the number of predicted classes.

The statistical approaches FOS and Sequoia display different behaviors on Datasets A and B displayed respectively on right and left panels of Figure 1. For the classification *α*-Other (green curves), the success rates are better for NMR (B) than for X-ray (A) datasets. This difference might come from *α* structures more regular than other secondary structure elements in NMR structures. Indeed, in NMR studies, the proton nuclei present in *β* strand regions are more difficult to assign than for other secondary structure elements.

For other classifications, the results are inverted as the success rates are better for X-ray (A) than for NMR (B) structures, especially in the presence of noise *η*_2_. The difference even goes up to 6% for classification All. The smaller success observed in the case of NMR solution structures is not surprising as the larger flexibility in solution, which reduces the precision of these structures and consequently hampers the learning procedure. In addition, as described in Section 2.8, the Dataset B is only used for testing purpose and not for training.

When only $C_{\alpha }$ atoms were included, the prediction of secondary structures (Fig. 1, lower panels) displays features similar to those observed when all atoms were included in the graph. In case where no noise was added to the angle/distance information, the *F*1-scores were the most decreased, but the decrease was bigger for FOS than for Sequoia. Overall, the prediction of *α* helix alone (green curves) keeps quite similar scores than in the case of all backbone heavy atoms were considered. There is a marked decrease of the success as soon as more than one type of secondary structure is considered. The $C_{\alpha }$ networks seems thus to have less discriminating features between different secondary structures than the all network of heavy backbone atoms. When the Datasets A (left panels) and B (right panels) are compared, the improvement for *α*-Other (green curves) in the Dataset B is similar that the one observed for all atoms. For the classification All, the proteins of Dataset B display significantly worse results than those of Dataset A.

### 3.2 Effect of degraded input

Supplementary Figure S3 displays the Sequoia results in the case of degraded input. Two cases were investigated: (i) the ablation of various percentages of atoms (left panels) and (ii) the number of considered neighbors in the graph (right panels). The rational for exploring these aspects is the presence of noise in all experimental techniques of structural biology. The reason for analyzing the aspect (ii) is rather the numerous protein structures for which regions are not visible due to various experimental problems described in Section 1.

The effect of degraded input was investigated by reducing randomly the number of residues in the graph (left panels) or by increasing the number of connected neighbors described by the hyperparameter *k* (right panels) introduced in Section 2.1. In the graph including all backbone heavy atoms, several percentages of residues ablation from the graph network were considered (Supplementary Fig. S3, upper left panel). It is remarkable that the prediction by Sequoia is reduced from <10% for all ablation levels smaller than 20%. For larger ablation levels, the success rate decreases strongly but, for the prediction of *α* or *β* elements, is mostly reduced of about 20% for an ablation level of 50%. The two Datasets A and B (continuous and dashed lines) display similar resistance to ablation for all predictions.

The influence of the hyperparameter *k*, defining for each residue, the number of neighbor residues connected by an edge in the graph, was also investigated (Supplementary Fig. S3, upper right panel). Hyperparameter values *k* in the range 3–5 have been explored in addition to the value of *k *=* *2 used in the previous analyses (Fig. 1). The predictions are more robust to the increase of *k* than they were to the ablation of residues. Sequoia displays improved success rates along the number of neighbors for all types of investigated predictions. As the neighbor residues are added to the graph basing only on a distance criterion, they are shared between residues close in the primary sequence and other far apart in the primary sequence. The increase of success rates observed when adding more neighbor residues, gives an insight that the generalized definition of ϕ and *ψ* proposed in this work, is quite efficient to decipher between residues close and far apart in the primary sequence. Indeed, the detection of secondary structure elements favor the residues close in the primary sequence to the detriment of the residues far apart in the primary sequence.

The effect of degraded input was also tested on the simplified network containing only atoms $C_{\alpha }$ (Supplementary Fig. S3, bottom panels). Concerning the random ablation of residues, the results on $C_{\alpha }$ graphs are quite similar (Supplementary Fig. S3, bottom left panel) to the results obtained on the backbone atom graph, with an overall reduction of scores of about 5% for ablation percentages up to 20%. For ablation percentages larger than 20%, the *α*-Other (green curves) prediction is much more affected than the predictions *β*-Other (black curves) and *α*-*β*-Other (cyan curves) predictions, which display relatively flat variations according to the increase of ablation. This might be related to the difference of geometry between an *α* helix and a straight line corresponding to a *β* region. Indeed, in a helix defined by points, the removal of points has a larger influence on the perception of the geometric figure than in a straight line. The influence of the hyperparameter *k* was also investigated (Supplementary Fig. S3, bottom right panel) for the graph containing only atoms $C_{\alpha }$. The observed trends were similar to those for the graph including all backbone heavy atoms. Nevertheless, the increase of *F*1 score is less marked and corresponds rather to a plateau of values. In addition, the efficiency of Sequoia was tested on the Dataset A’, extracted from the server PISCES (Wang and Dunbrack, 2003) with structures determined at a resolution between 3 and 5 Å, and with *R* factors worse than 0.25. The *F*1 scores obtained by Sequoia on this Dataset (Supplementary Table S1A) are quite close from the one obtained on the Dataset A.

### 3.3 Sequoia and other approaches for determining secondary structure

The Sequoia results have been put in parallel with various alternative approaches for secondary structure prediction. The efficiency of Sequoia for the prediction of secondary structure was compared to the software PSIPRED (Jones, 1999), which takes as only input the protein sequence. PSIPRED 4.02 was run on the proteins of Dataset A, and *F*1 scores of Sequoia have been calculated comparing the Sequoia output to DSSP and PSIPRED outputs (Supplementary Table S1B). The *F*1 scores obtained using PSIPRED outputs are smaller than the ones obtained using DSSP: the difference is in the range 5–7 for the all-atom systems and is three for the C*α* systems. This proves that the geometric input used by Sequoia, although it does not contain sequence information, produces information closer to DSSP output than to PSIPRED output.

The human proteins present in the database of AlphaFold models at alphafold.ebi.ac.uk has been screened to get the entries containing structures present in the Dataset A. These AlphaFold models were downloaded from the EBI database and the domain structures extracted. These 312 domain models were processed by Sequoia and their *F*1 scores with respect to DSSP were compared to the *F*1 score obtained on the corresponding domains in the Dataset A. The *F*1 scores are similar (Supplementary Table S1C) to these previously obtained on Dataset A whatever all atoms or only the atoms C*α* are considered.

The software STRIDE (Frishman and Argos, 1995) has been run on proteins of Dataset A and the secondary structure elements extracted. The Sequoia prediction have been then compared to the STRIDE prediction, and similar results were obtained when using DSSP as comparison check (Supplementary Table S1D).

### 3.4 Positions of sequoia erroneous predictions

The error cases in Sequoia prediction were examined for Dataset A in the absence of noise (Supplementary Fig. S4, upper panel). For each erroneously predicted residue, the distance of the residue to the extremity of the corresponding secondary structure element was determined. For classifications *α*-Other and *β*-Other, a large majority of the erroneous predictions (*w *=* *2) were located at the limits within the two first or the two last residues of a secondary structure element, most of them being the first or the last residue (*w *=* *1). These erroneous predictions are the sign of different points of view on the limits of secondary structure elements. DSSP handles a discrete classification, whereas Sequoia is sensitive to the geometrical deformations close to the limits, which leads to exclude the limit residues from the detection of the element. If one would exclude the limit residues from the initial definition of the secondary structure element, the success rates in Figure 1 would increase for Sequoia from 94.4 up to about 97% for the prediction *α*-Other.

The positions of the residues erroneously assigned to secondary structure elements in a $C_{\alpha }$ graph (Supplementary Fig. S4, bottom panel), displays a quite striking difference from the predictions realized in the graph including backbone heavy atoms. Indeed, the erroneous *β*-Other predictions are in majority located at the extremity of the *β* strands, but in a lesser extent that for the graph built from backbone heavy atoms (Supplementary Fig. S4). At the contrary, the erroneous *α*-Other predictions are more often located at the extremity of the *α* helices than in the all-atom graph. This difference of behavior between the graph of Carbons *α* and the all-atom graph is related to the differences in the geometry of a helix and a straight line mentioned above.

### 3.5 Examples of sequoia use

Some examples of Sequoia predictions are given for three proteins displaying only *α* helices, only *β* strands or both types of secondary structures (Supplementary Fig. S5). The *α* helices and *β* strands are in good agreement with the DSSP predictions. The missing residues in the prediction of secondary structure elements are mostly located at the extremities of the elements in agreement with the previous analyses of Supplementary Figure S4.

The efficiency of Sequoia prediction was also tested on $C_{\alpha }$ positions determined using Deeptracer (Pfab *et al.*, 2021; Si *et al.*, 2020) on three EM maps obtained from the EMDB (www.ebi.ac.uk/pdbe/emdb/): EMD-23927 (Hoq *et al.*, 2021), EMD-30915 (Liu *et al.*, 2021) and EMD-30942 (to be published). These entries were chosen as they correspond to different protein complexes (affinity captured human p97 hexamer, *Salmonella flagella* MS-ring protein FliF 1-456, apo spike protein of SARS-CoV2). They were obtained by single particle reconstruction and correspond to medium-resolution data, for which the determination of atomic positions is not straightforward. The resolutions for the entries EMD-23927, EMD-30915 and EMD-30942 were respectively of: 4.22, 3.45 and 4.46 Å, and no corresponding PDB entry has been described in EMDB for these data.

**Fig. 2.**
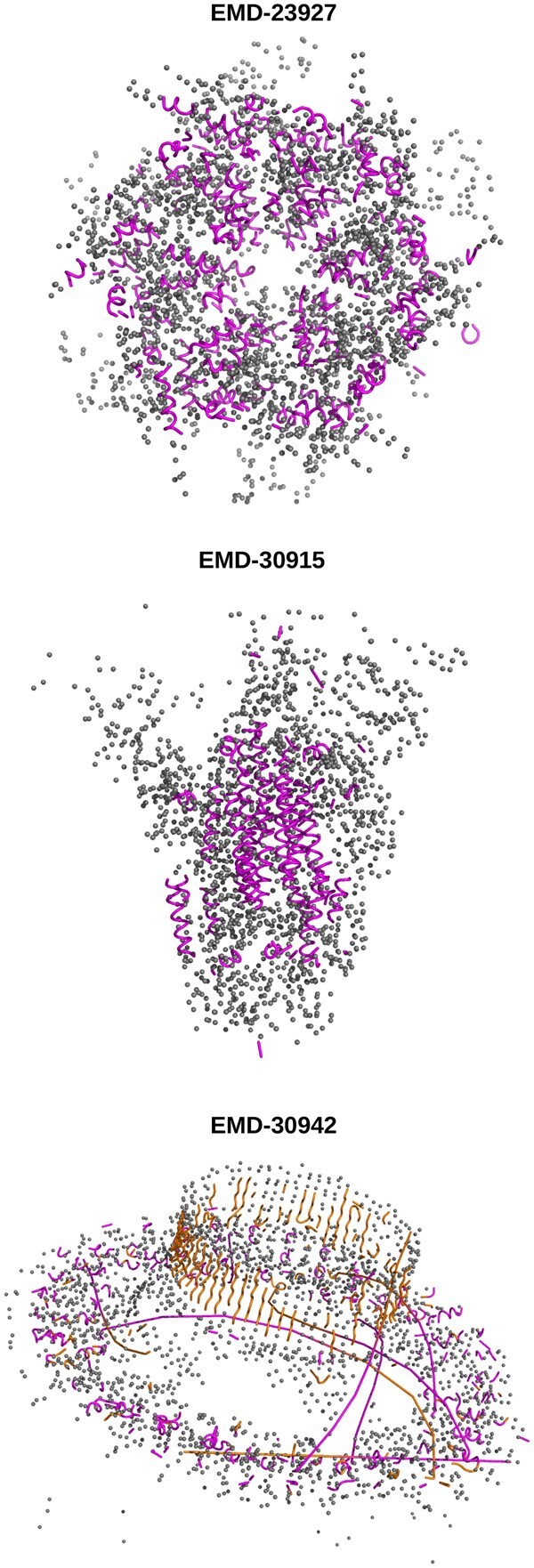
Results of Sequoia on outputs of the Deeptracer Web site (Si *et al.*, 2020) deeptracer.uw.edu. The Sequoia prediction *α*-Other was run on EMD-23927 and EMD-30915 whereas the Sequoia prediction *α*-*β*-Other was run on EMD-30942. In each panel, the predicted *α* helices and *β* strands are drawn as cartoon, and other residues as gray spheres, and are labeled by the corresponding entry in Abbott *et al.* (2018). The detected *α* helices are colored in magenta and the *β* strands in orange. The structure images were produced using pymol (DeLano, 2002)

The EM maps were uploaded to the Deeptracer Web server (deeptracer.uw.edu/home) and the deep-learning prediction of atoms positions was run using the default parameters. The output containing only $C_{\alpha }$ atoms was downloaded and given to the Sequoia prediction tool trained on the database of $C_{\alpha }$ graphs with the classifications *α*-Other and *α*-*β*-Other. The results of the prediction are displayed in Figure 2. The predicted *α* helices and *β* strands are drawn in cartoon whereas the residues predicted to belong to the classification Other are drawn as spheres. Sequoia is able to catch quite a number of the secondary structure elements expected in these structures.

The backbone tracing results obtained by Deeptracer (Pfab *et al.*, 2021; Si *et al.*, 2020) on the selected entries from the EMDB were compared to the results produced by Sequoia, by calculating the number of secondary structure elements detected and their average lengths (Supplementary Table S2). Sequoia detects larger number of elements with shorter lengths, which is the sign of a larger fragmentation of the elements. This tendency is not surprising as Deeptracer makes use of the vertex information provided by EM maps and follows a step-by-step prediction path whereas Sequoia just uses as input the sparse information coming from the C*α* positions.

## 4 Discussion

The main outcome of this work is to propose a method for predicting the secondary structure elements of proteins using as input the distances between atoms and not requiring the knowledge of residue succession in protein sequence. To the best of our knowledge, this is the first time in the literature that secondary structures are predicted in such a frame. We showed above that this approach was made possible by a generalization of dihedral backbone angles ϕ and *ψ* for (i) the case of couples of residues, covalently bonded in the protein sequence or not, as well as for (ii) the case of a $C_{\alpha }$ atoms graph.

The type of neural network used for the Sequoia prediction is also an innovative aspect of the approach, as it is a MPN. Although MPN approaches have already been used in the context of ligand docking (Fout *et al.*, 2017; Zhao *et al.*, 2021; Zhu *et al.*, 2020), this type of neural network is used here for the first time in the context of protein structure prediction. In order to apply the MPN approach, we have constructed a graph on the protein residues in which the existence of an edge depends only on a threshold distance between the residues vertices, and not on their involvement in a covalent bond and is thus independent from the sequence information. This approach can exploit an essential advantage of MPN methods when dealing with fragments of protein structures, as it is the case if disordered regions of the protein are not observed, or if one deals with medium-resolution EM maps.

Sequoia performs better than FOS, and is resistant to noise. The classifications producing the best success rates are *α*-Other, *β*-Other and *α*-*β*-Other, in agreement with the knowledge on the ranges of dihedral angles in proteins. The three classifications *α*-Other, *β*-Other and *α*-*β*-Other, obtained by Sequoia, are successful at percentages mostly larger than 80% even for the less precise Dataset B formed with NMR structures. Sequoia approach is also remarkably resistant to the ablation of protein residues and to the variation of distance threshold between residues.

The examination of individual residue errors in Sequoia revealed that most of these errors are located within the two first or last residues of the considered secondary structure elements. The origin of such errors arises from the choice of the method DSSP (Kabsch and Sander, 1983) as reference for validating Sequoia. Indeed, DSSP implements a discrete classification of residues among secondary structures in which the prediction jumps from one to another value at the limits of secondary structure elements, without continuous interpolation. Such discontinuity disagrees obviously with the protein structure variations, which occur continuously along the protein backbone, as shown in the approach screwfit (Calligari and Kneller, 2012), based on a modeling of the protein backbone in terms of a curve with intrinsic torsion.

Sequoia represents also a step toward a coarse-grained perspective of the interval iBP approach (Lavor *et al.*, 2012; Liberti *et al.*, 2014). Indeed, iBP, as well as Sequoia, is based on the use of distances and angles (Worley *et al.*, 2018) inputs, and was up-to-now, an algorithm basing the protein structure determination on a tree building, each tree level corresponding to atoms. With the help of Sequoia, it should be now possible to consider the replacement of certain groups of atoms by secondary structure elements. In that way, the tree will be simplified and the combinatorial problems due to algorithm complexity reduced.

Sequoia displays results on a graph containing only atoms $C_{\alpha }$, which are similar than the results obtained considering all backbone heavy atoms. Unsurprisingly, the reduced input information produces a decrease of the *F*1 scores. Nevertheless, Sequoia displays a reasonable robustness with respect to the reduction of the information from the molecular graph. Similarly, Sequoia shows constant success rates or even improvements when the complexity of the graph is increased by increasing the number of neighbors described by the hyperparameter *k*.

As Sequoia is able to predict secondary structure elements from the positions of atoms C*α*, it could generate on the fly cartoon representation of secondary structure. This would be of great help for the 3D visualization of low resolution structures.

One can also notice that the prediction of secondary structure elements by Sequoia permits to assign residues to the same element. This provides the sequence succession information within such elements.

In cryo EM, the detection of secondary structure elements in the medium-resolution EM maps is a fundamental step for connecting EM signal to structural information. The analysis of $C_{\alpha }$ graphs performed here have some relationship to the EM maps as the $C_{\alpha }$ atoms can be considered as a simplified description of the residue electronic density or of the EM map voxel.

## Funding

This work was supported by the ANR project Project-ANR-19-CE45-0019 (multiBioStruct) as well as by CNRS, Institut Pasteur and Ecole Polytechnique.

*Conflict of Interest*: none declared. 

## Supplementary Material

vbab038_Supplementary_Data
