# Supplementary material for: Secondary structure assignment of proteins in the absence of sequence information
Source: Bioinform Adv. 2021 Nov 29;1(1):vbab038. doi: 10.1093/bioadv/vbab038 (PMC9710659; doi:10.1093/bioadv/vbab038)
Supplement: vbab038_Supplementary_Data [file vbab038_supplementary_data.pdf]

# Supplementary material: Secondary structure assignment of proteins in the absence of sequence information

Sammy Khalife [1,a], Thérèse E. Malliavin [2] and Leo Liberti [1]

[1] *LIX, CNRS, Ecole Polytechnique, Institut Polytechnique de Paris, 91128, Palaiseau, France*

[2] *Institut Pasteur, Université de Paris, CNRS UMR3528, F-75015 Paris, France*

[a] New affiliation: Department of Applied Mathematics and Statistics, Johns Hopkins University

**Email**: khalife@lix.polytechnique.fr (New affiliation: khalife.sammy@jhu.edu)

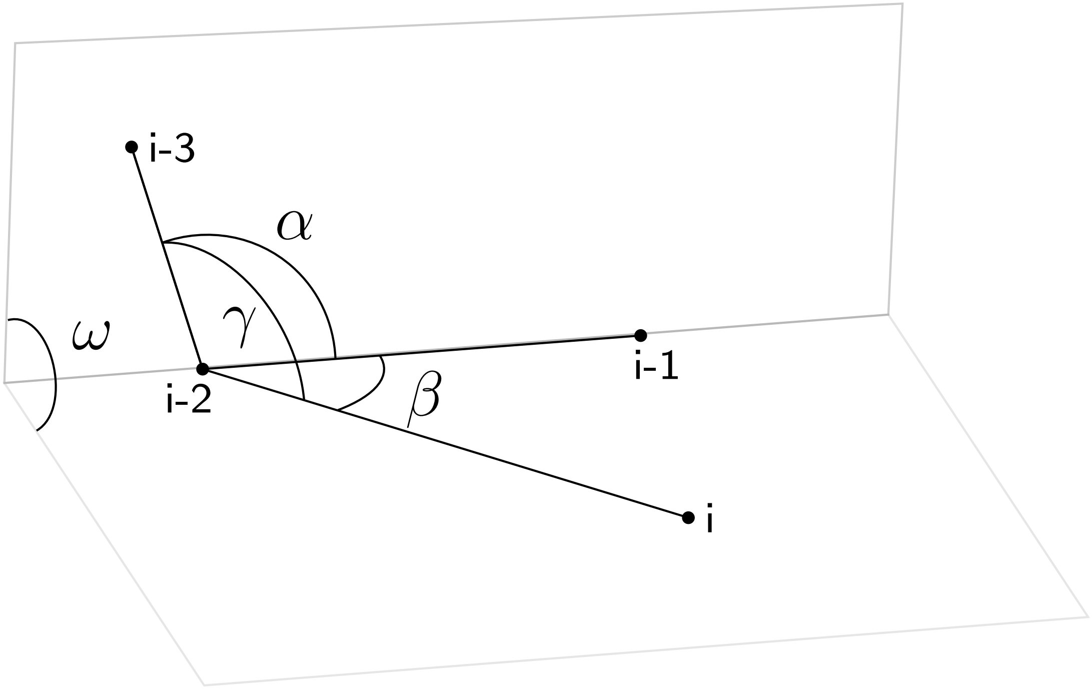


Figure S1: Two planes made by a group of four atoms $i-3$, $i-2$, $i-1$ and $i$. $\omega$ represents the dihedral angle between the two planes. The angles $\alpha$, $\beta$ and $\gamma$ are calculated from the distances between atoms using Eq 2 in the main text.

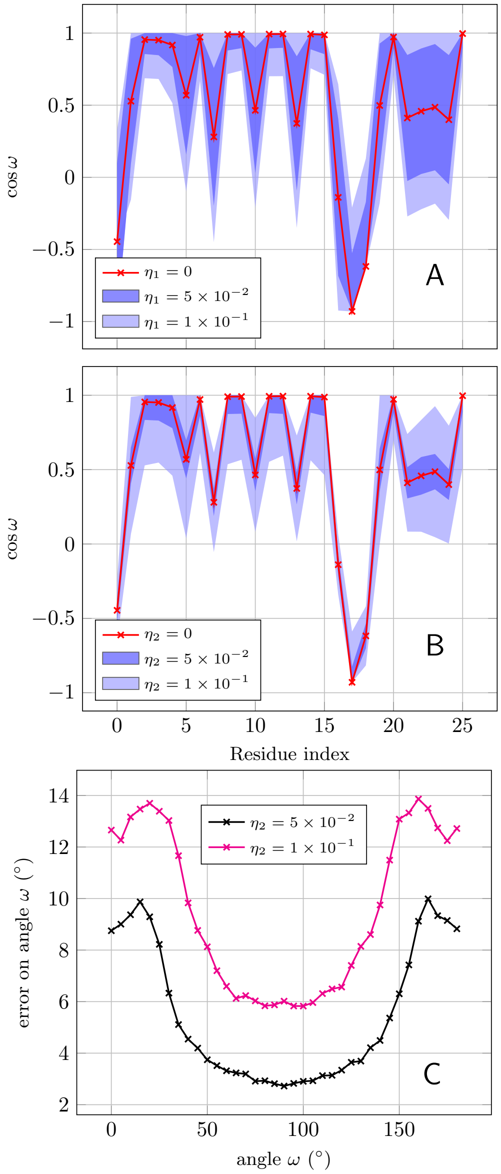


Figure S2. A, B: Impact of noise on the cosine of dihedral angle $\omega$ on the 25 first residues of the protein 1M22 of PDB. The noise level is defined by the parameters: (A) $\eta_1$ (Å) on the distances and (B) $\eta_2$ on $\cos\omega$. The red curves defines the true $\cos\omega$ values, and the darkcyan and palecyan surfaces define the variations of $\cos\omega$ according to the noise level. C: Variation of the error on the angle $\omega$ with respect to the value of $\omega$. The average error is calculated by Monte Carlo using 1000 realisation of Gaussian noise. The error on $\omega$ is computed as the standard deviation of $\omega - \arccos(\cos(\omega) + \varepsilon)$, where $\varepsilon = \mathcal{N}(0, \eta_2)$.

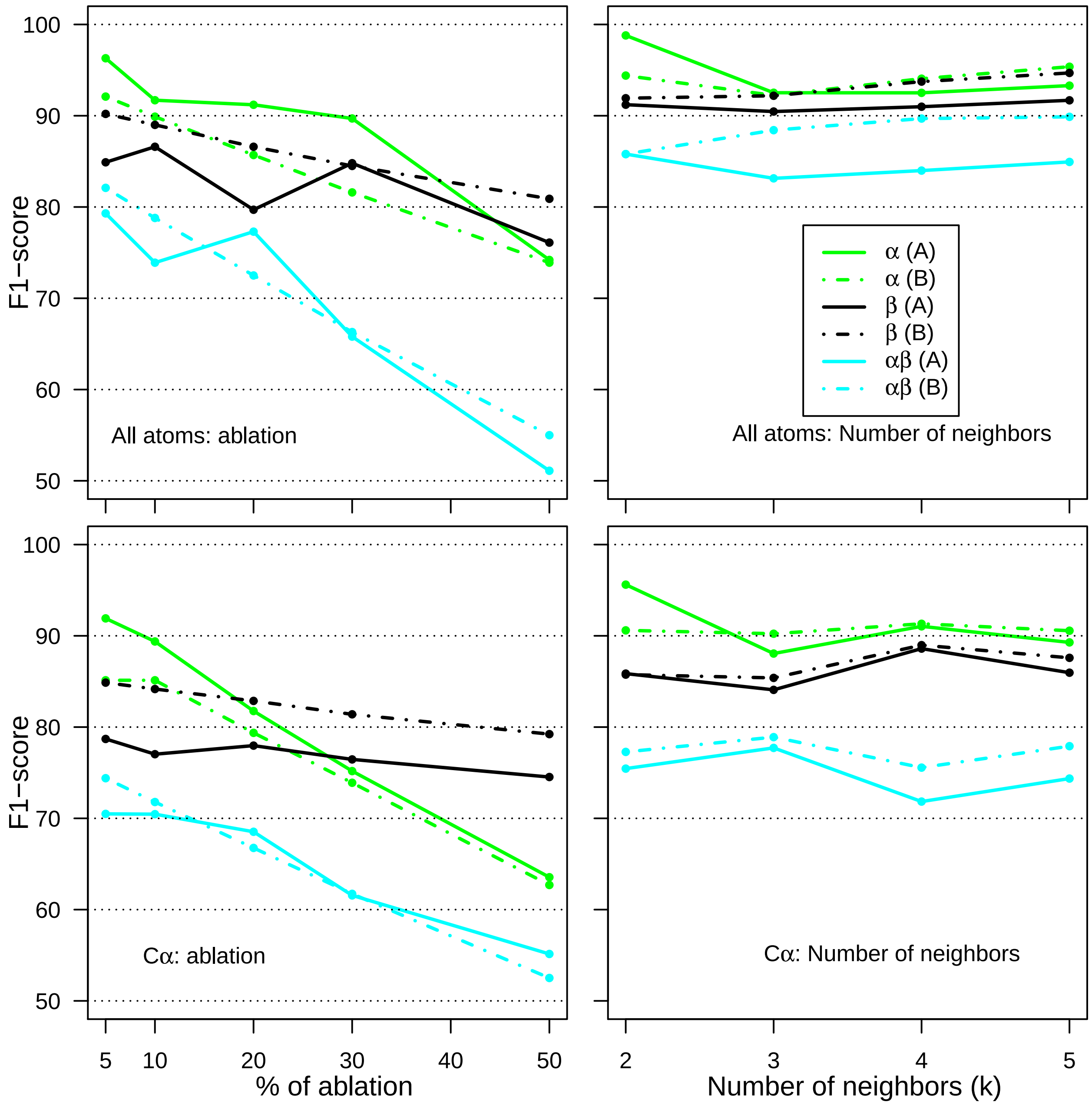


Figure 2: *

Figure S3. Effect of input degradation. The Sequoia predictions (F1-score) are displayed for all atoms (upper panels) and atoms $C_\alpha$ (lower panels) and for percentages of ablation (left panels) and number of considered neighbors in the graph (right panels). The predictions are displayed for datasets A (continuous lines) and B (dotdash lines). The predictions are plotted according to the percentage of ablation (left panels) or to the number of considered neighbors in the graph (right panels).

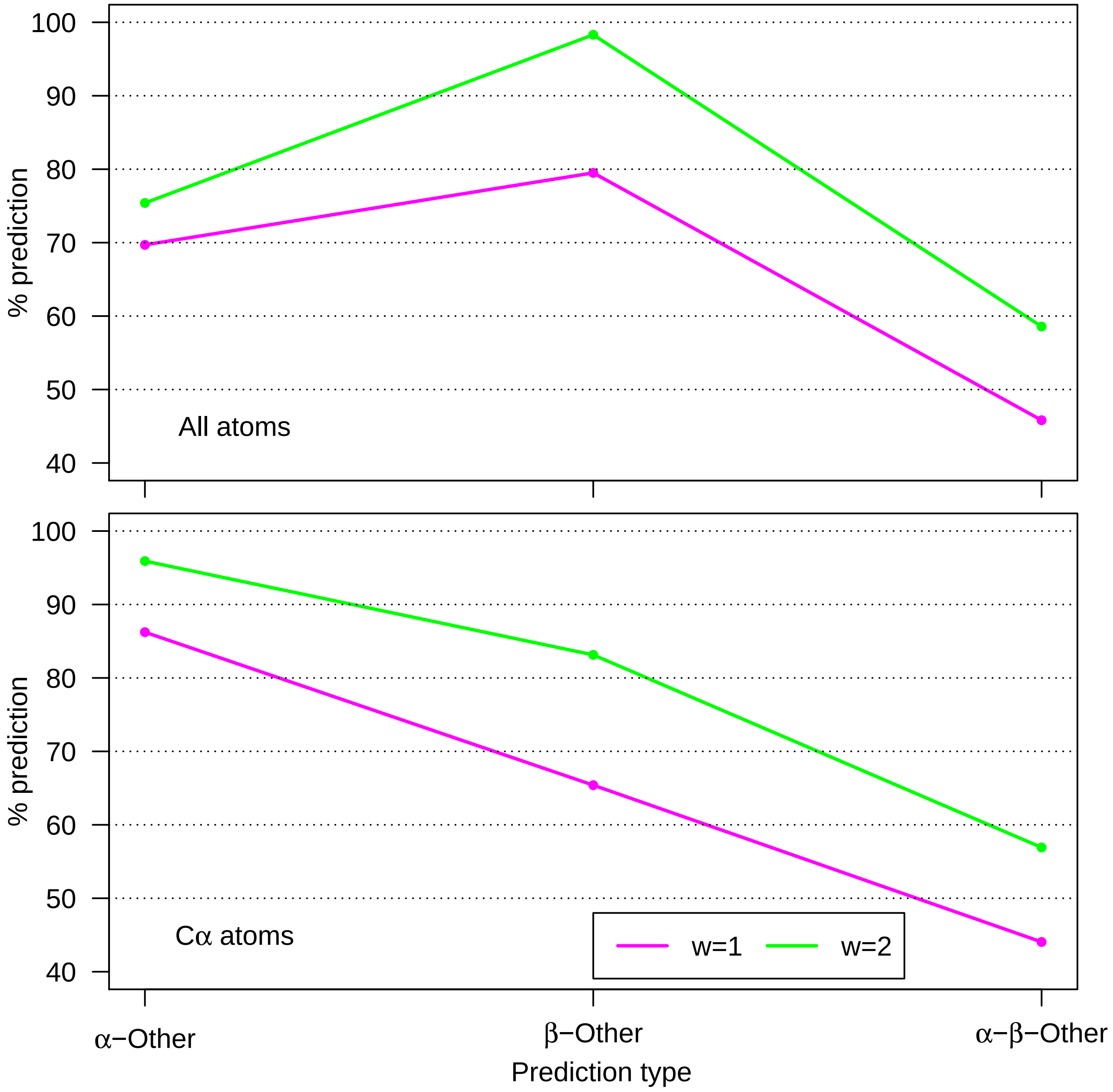


Figure 3: *

Figure S4. Position of Sequoia errors in secondary structure elements. The percentages of erroneous Sequoia predictions within the first or last residue of the element ($w = 1$, magenta curve) or within the two first or two last residues of the element ($w = 2$, green curve) are plotted according to the type of prediction ($\alpha$-Other, $\beta$-Other, $\alpha$-$\beta$-Other).

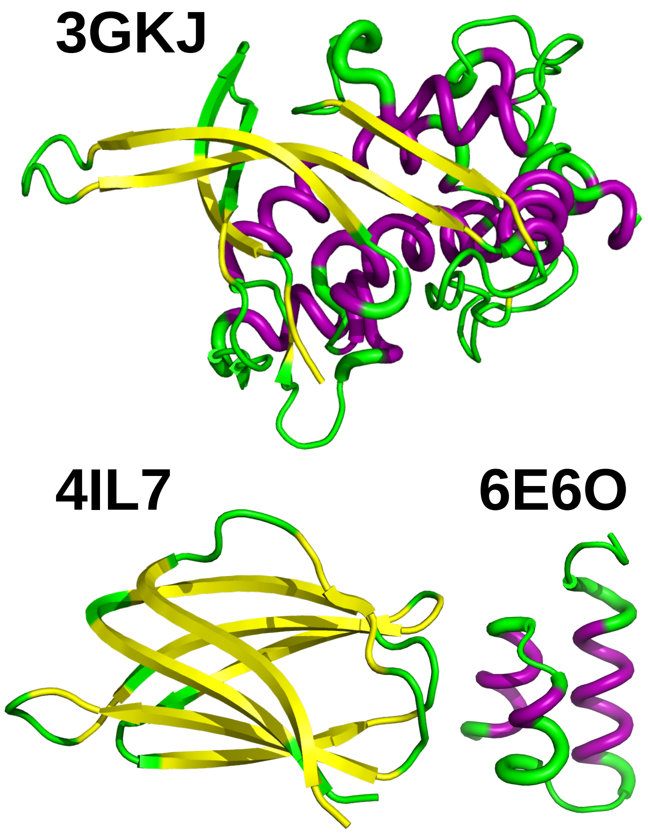


Figure 4: *
Figure S5. Three examples of structures taken from dataset A. The structures are drawn in cartoon according to the DSSP[1] predictions whereas the regions predicted by Sequoia as $\alpha$ helices or $\beta$ strands are colored in purple and yellow. The structures are labeled with the names of the corresponding PDB entries. The structure images were produced using pymol.[2]

Table S1: Comparison of the Sequoia F1 scores for the classification $\alpha$-$\beta$-Other obtained for various cases: A. the use of the low resolution Dataset A' extracted from the server PISCES [3] with structures determined at a resolution between 3 and 5 Å, and with R factors worse than 0.25. B. the use of PSIPRED.[4] C. the use of the dataset built from the 312 human protein chains extracted from the database of AlphaFold models at `alphafold.ebi.ac.uk`. D. The use of the STRIDE[5] for protein structure predictions. Depending on the case, the Sequoia results are compared to DSSP,[1] STRIDE[5] and PSIPRED[4] results.

| | | |
|---|---|---|
| A. Low resolution | | |
| noise | score all atoms | score C$\alpha$ |
| 0.0 | 83 | 58 |
| 0.05 | 83 | 58 |
| 0.1 | 82 | 58 |
| B. Comparison PSIPRED | | |
| noise | score all atoms versus DSSP | score all atoms versus PSIPRED |
| 0.0 | 85 | 78 |
| 0.05 | 85 | 78 |
| 0.1 | 83 | 78 |
| noise | score C$\alpha$ versus DSSP | score C$\alpha$ versus PSIPRED |
| 0.0 | 53 | 50 |
| 0.05 | 53 | 50 |
| 0.1 | 53 | 50 |
| C. AlphaFold | | |
| noise | PDB domains | AlphaFold domains |
| 0.0 | 85 | 84 |
| 0.05 | 85 | 84 |
| 0.1 | 83 | 83 |
| D. Comparison STRIDE/DSSP | | |
| noise | score all atoms versus DSSP | score all atoms versus STRIDE |
| 0.0 | 85 | 85 |
| 0.05 | 85 | 85 |
| 0.1 | 83 | 84 |
| noise | score C$\alpha$ versus DSSP | score C$\alpha$ versus STRIDE |
| 0.0 | 54 | 55 |
| 0.05 | 54 | 55 |
| 0.1 | 54 | 55 |

Table S2: Comparison of the secondary structure elements predicted by Deeptracer[6,7] and by Sequoia on the cryo-EM maps displayed in the Figure 2 of the main text.

| System | Prediction | Number of $\alpha$-helices | average size of $\alpha$-helices | Number of $\beta$-strands | average size of $\beta$-strands |
|---|---|---|---|---|---|
| EMD23927 | Deeptracer | 143 | 11 residues | 55 | 4.2 residues |
| EMD23927 | Sequoia | 157 | 5 residues | 34 | 2 residues |
| EMD30915 | Deeptracer | 45 | 13.7 residues | 77 | 4.5 residues |
| EMD30915 | Sequoia | 49 | 8 residues | 41 | 3 residues |
| EMD30942 | Deeptracer | 103 | 10.9 residues | 111 | 1.3 residues |
| EMD30942 | Sequoia | 155 | 2 residues | 89 | 3 residues |

Dataset A : Dataset A is composed of 3621 protein X-ray crystallographic structures downloaded from the server PISCES.[3] These high quality structures correspond to a set of Protein Data Bank (PDB)[8] entries for which structures have been determined at a resolution better than 1.6 Å, and with R factors better than 0.25. The set of PDB entries and protein chains present in dataset A has been chosen[3] in order that the percentage of sequence identity between any pair of chains is smaller than 20%, to avoid statistical bias on the protein sequences.

1A62(A), 1BX7(A), 1CCW(D), 1DJ0(A), 1EDM(B), 1F46(A), 1FYE(A), 1GK7(A), 1GUT(A), 1H2C(A), 1HZ4(A), 1IDP(A), 1J2J(B), 1JF8(A), 1JO0(B), 1K5N(A), 1KOE(A), 1L3K(A), 1LQT(A), 1M2D(A), 1MN8(A), 1N62(C), 1NTH(A), 1O7I(A), 1OK0(A), 1P9H(A), 1Q5Y(A), 1QS1(A), 1R6J(A), 1RK6(A), 1RYO(A), 1SEN(A), 1T1G(A), 1T92(A), 1TZP(A), 1UCS(A), 1USE(A), 1V0W(A), 1VBW(A), 1VKE(A), 1VRZ(A), 1AH7(A), 1BYI(A), 1CXQ(A), 1DP7(P), 1EGW(A), 1F9V(A), 1G2R(A), 1GK9(B), 1GV9(A), 1H32(A), 1HZT(A), 1IFR(A), 1J3A(A), 1JFB(A), 1JOV(A), 1K7C(A), 1KQ6(A), 1L6R(A), 1LQV(A), 1M4L(A), 1MNN(A), 1N8V(A), 1NU0(A), 1O7J(A), 1OQJ(A), 1P9I(A), 1Q6Z(A), 1QV1(A), 1R6X(A), 1RKI(A), 1S1D(A), 1SFS(A), 1T1U(A), 1T9I(A), 1U07(A), 1UFY(A), 1USM(A), 1V2X(A), 1VCC(A), 1VKK(A), 1VYI(A), 1AHO(A), 1C0P(A), 1CY5(A), 1DS1(A), 1EKQ(A), 1FCQ(A), 1G2Y(A), 1GKM(A), 1GVP(A), 1H4X(A), 1I1W(A), 1INL(A), 1J3W(A), 1JG1(A), 1JX6(A), 1K7J(A), 1KQF(C), 1L9L(A), 1LS1(A), 1M55(A), 1MUN(A), 1NC7(A), 1NUY(A), 1O98(A), 1OU8(B), 1PKH(A), 1Q7L(B), 1QV9(A), 1R7J(A), 1RKU(A), 1S29(A), 1SN9(A), 1T3Y(A), 1TBF(A), 1U2H(A), 1UGI(A), 1UTG(A), 1V4P(A), 1VD6(A), 1VL7(A), 1VYK(A), 1ATG(A), 1C1D(A), 1D5T(A), 1E29(A), 1ELK(A), 1FCY(A), 1G3P(A), 1GMU(A), 1GWE(A), 1H97(B), 1I24(A), 1IO0(A), 1J77(A), 1JHJ(A), 1JY2(O), 1KAF(A), 1KQP(A), 1L9X(A), 1LU4(A), 1M9Z(A), 1MUW(A), 1NG6(A), 1NWZ(A), 1OAI(A), 1OW4(A), 1PMH(X), 1QFT(A), 1QW2(A), 1R9L(A), 1ROC(A), 1S2O(A), 1SQS(A), 1T61(A), 1TKE(A), 1U7G(A), 1UGX(B), 1UUY(A), 1V5I(B), 1VE4(A), 1VLY(A), 1VYR(A), 1B0B(A), 1C1K(A), 1D8W(A), 1E58(A), 1ES5(A), 1FIU(A), 1G61(A), 1GMX(A), 1GWM(A), 1HPG(A), 1I27(A), 1IQZ(A), 1J98(A), 1JI7(A), 1JYK(A), 1KGD(A), 1KT6(A), 1LC0(A), 1LUC(A), 1MC2(A), 1MWQ(B), 1NKD(A), 1NXM(A), 1OCY(A), 1P1X(A), 1PO5(A), 1QG8(A), 1QW9(B), 1RA0(A), 1RP0(A), 1S3C(A), 1SVS(A), 1T6F(A), 1TP6(A), 1U7I(A), 1UKF(A),

1UWC(B), 1V5V(A), 1VH5(A), 1VMG(A), 1VZM(A), 1BGF(A), 1C4Q(D), 1DCS(A), 1E5K(A), 1ET1(A), 1FM0(E), 1G6G(A), 1GNL(A), 1GXM(A), 1HQ1(A), 1I2T(A), 1IRQ(A), 1JB3(A), 1JKE(A), 1K3X(A), 1KJQ(A), 1KWF(A), 1LC5(A), 1LWB(A), 1MG7(A), 1MY7(A), 1NKI(A), 1NYC(A), 1OD3(A), 1P4C(A), 1PP0(A), 1QGV(A), 1QWG(A), 1RDQ(I), 1RTQ(A), 1S9R(A), 1SX5(A), 1T6U(A), 1TQG(A), 1U84(A), 1UNQ(A), 1UWK(A), 1V6P(A), 1VHN(A), 1VMH(A), 1W0H(A), 1BKR(A), 1C5E(A), 1DD9(A), 1E7L(A), 1EUW(A), 1FO8(A), 1G6X(A), 1GP0(A), 1GY7(A), 1HW1(A), 1I4U(A), 1IUQ(A), 1JBE(A), 1JL1(A), 1K4I(A), 1KKO(A), 1KWG(A), 1LLF(A), 1M15(A), 1MJ5(A), 1N08(A), 1NLQ(A), 1NZ0(A), 1OF8(B), 1P5Z(B), 1PSR(A), 1QLW(A), 1QWO(A), 1RFY(A), 1RTT(A), 1S9U(A), 1SXR(A), 1T7F(B), 1TT8(A), 1U8V(C), 1UOY(A), 1UZ3(A), 1V70(A), 1VHT(A), 1VP8(A), 1W0N(A), 1BTE(A), 1C7K(A), 1DFM(A), 1EAQ(A), 1EVL(A), 1FSG(C), 1GCI(A), 1GPP(A), 1GYX(A), 1HXI(A), 1I5G(A), 1IX9(A), 1JEK(A), 1JNI(A), 1K4N(A), 1KMT(A), 1KYF(A), 1LMI(A), 1M1F(A), 1MK0(A), 1N13(B), 1NNL(A), 1NZJ(A), 1OI0(A), 1P6O(A), 1PZ4(A), 1QOW(D), 1R29(A), 1RG8(B), 1RWH(A), 1SAU(A), 1SZ7(A), 1T7M(B), 1TU9(A), 1UCD(A), 1US0(A), 1UZK(A), 1V7W(A), 1VJU(A), 1VQS(A), 1W1H(A), 1BTK(A), 1CC8(A), 1DG6(A), 1EB6(A), 1EZG(A), 1FT5(A), 1GJ7(A), 1GQI(A), 1H16(A), 1HYO(A), 1ID0(A), 1J0P(A), 1JET(A), 1JNR(A), 1K5C(A), 1KNM(A), 1KYH(A), 1LNI(A), 1M22(A), 1MKK(A), 1N3L(A), 1NNX(A), 1O06(A), 1OI7(A), 1P9G(A), 1Q0R(A), 1QQF(A), 1R6D(A), 1RJU(V), 1RYL(A), 1SBY(A), 1SZH(A), 1T8K(A), 1TUA(A), 1UCR(A), 1US5(A), 1V05(A), 1V7Z(A), 1VK1(A), 1VR7(A), 1W23(A), 1W4S(A), 1WDD(A), 1WNA(A), 1WZD(A), 1XBI(A), 1XSZ(A), 1YBK(A), 1Z0W(A), 1ZCE(A), 2A0B(A), 2AKZ(A), 2AYD(A), 2BDR(A), 2BMO(B), 2C2U(A), 2CC6(A), 2CIU(A), 2CXN(A), 2DDX(A), 2DTJ(A), 2E5F(A), 2EPL(X), 2F5T(X), 2FCO(A), 2FQ3(A), 2G30(A), 2GKE(A), 2GUD(B), 2H8E(A), 2HLY(A), 2I3D(A), 2IAY(A), 2IMJ(A), 2IZX(A), 2J9W(A), 2JHF(B), 2NN5(A), 2NXV(A), 2O90(A), 2OFK(A), 2OOA(A), 2OYO(A), 2P51(A), 2PEZ(A), 2PQ8(A), 2PYX(B), 2QFE(A), 2QPX(A), 2R0X(A), 2RAF(B), 2RIL(A), 2UYT(A), 2V89(A), 2VCL(A), 2VPB(B), 2W15(A), 2W5Q(A), 2WDS(A), 2WNK(A), 2WUH(B), 2X32(A), 2X5Y(A), 2XHI(A), 2XRH(A), 1W53(A), 1WER(A), 1WPA(A), 1X0T(A), 1XD3(A), 1XUB(A), 1YD0(A), 1Z2N(X), 1ZHV(A), 2A26(A), 2AML(A), 2B0A(A), 2BF9(A), 2BO9(B), 2C3V(A), 2CCQ(A), 2CJT(A), 2CYJ(A), 2DE3(A), 2DVM(A), 2E6F(A), 2ERF(A), 2F60(K), 2FCT(A), 2FR5(A), 2G3R(A), 2GKG(A), 2GUH(A), 2H8G(A), 2HOX(D), 2I49(A), 2IBD(A), 2INW(A), 2J1V(A), 2JAE(B), 2JIS(A), 2NNU(B),

2O0A(A), 2O9S(A), 2OFZ(A), 2OPC(A), 2OZH(A), 2P5K(A), 2PGN(A), 2PR7(A), 2Q2F(A), 2QGU(A), 2QRL(A), 2R16(A), 2RBD(B), 2RKV(A), 2V1M(A), 2V8F(C), 2VE8(A), 2VPT(A), 2W1J(A), 2W6A(A), 2WE5(B), 2WNP(F), 2WUJ(A), 2X3H(B), 2X9G(A), 2XIO(A), 2XRY(A), 1W5Q(A), 1WFB(A), 1WPU(A), 1X1K(F), 1XDN(A), 1Y1P(A), 1YFQ(A), 1Z2U(A), 1ZI8(A), 2A35(A), 2ANX(A), 2B3G(B), 2BFD(B), 2BOG(X), 2C60(A), 2CCV(A), 2CKK(A), 2CZL(A), 2DKJ(A), 2DWU(A), 2E7Z(A), 2EV1(A), 2F62(A), 2FCW(B), 2FRG(P), 2G7O(A), 2GKP(A), 2GUI(A), 2HBA(A), 2HP0(A), 2I53(A), 2IBN(A), 2IP6(A), 2J2J(A), 2JC9(A), 2JKU(A), 2NQW(A), 2O0M(A), 2O9U(X), 2OHW(A), 2OPL(A), 2OZT(A), 2P6W(A), 2PHN(A), 2PRV(A), 2Q3T(A), 2QIP(A), 2QSB(A), 2R2Z(A), 2RBK(A), 2RL8(B), 2V33(A), 2V8T(B), 2VFO(A), 2VQ2(A), 2W1R(A), 2W7A(A), 2WFI(A), 2WOL(A), 2WW6(A), 2X3M(A), 2XBG(A), 2XJ4(A), 2XTP(A), 1W5R(A), 1WHI(A), 1WS8(A), 1X6I(B), 1XG0(C), 1Y43(B), 1YLX(A), 1Z3E(B), 1ZK5(A), 2A3N(A), 2AP3(A), 2B4H(A), 2BHU(A), 2BRF(A), 2C61(A), 2CDC(B), 2CNQ(A), 2CZS(A), 2DKO(B), 2DXA(A), 2EAB(A), 2EW0(A), 2F69(A), 2FGQ(X), 2FSQ(A), 2G7S(A), 2GLZ(A), 2GUV(A), 2HC1(A), 2HS1(A), 2I5U(A), 2IC2(A), 2IT2(A), 2J43(A), 2JCB(A), 2JLI(A), 2NR7(A), 2O1Q(A), 2OB3(A), 2OIZ(D), 2OQZ(A), 2P0N(A), 2P8I(A), 2PKF(A), 2PRX(A), 2Q5C(A), 2QJW(D), 2QSK(A), 2R31(A), 2RDQ(A), 2TNF(A), 2V3G(A), 2V9L(A), 2VFR(A), 2VQP(A), 2W31(A), 2W7Z(A), 2WFW(A), 2WOY(A), 2WWE(A), 2X46(A), 2XDW(A), 2XJP(A), 2XTS(A), 1W66(A), 1WHZ(A), 1WTJ(A), 1X6Z(A), 1XLQ(A), 1Y6X(A), 1YN3(A), 1Z3X(A), 1ZKE(A), 2A6Z(A), 2APJ(A), 2B82(A), 2BK9(A), 2BSY(A), 2C71(A), 2CDP(A), 2COV(D), 2D1S(A), 2DLB(A), 2DXU(A), 2EGV(A), 2F01(A), 2F9H(A), 2FHZ(B), 2FUL(A), 2G8S(A), 2GOM(A), 2GXQ(A), 2HEU(A), 2HUH(A), 2I5V(O), 2IC6(A), 2IU5(A), 2J5G(A), 2JDA(A), 2MCM(A), 2NRR(A), 2O2X(A), 2OB5(A), 2OKF(A), 2OV0(A), 2P0S(A), 2P9W(A), 2PLX(B), 2PU3(A), 2Q9K(A), 2QJZ(B), 2QSW(A), 2R5O(A), 2RE2(A), 2TPS(A), 2V3I(A), 2V9V(A), 2VH3(A), 2VWS(A), 2W39(A), 2W8T(A), 2WH6(A), 2WQ4(A), 2WWX(B), 2X49(A), 2XEP(A), 2XN6(B), 2XTT(A), 1W6S(B), 1WKQ(A), 1WVF(A), 1X7D(A), 1XMK(A), 1Y8A(A), 1YNP(B), 1Z67(A), 1ZL0(A), 2ABS(A), 2ARC(A), 2B97(A), 2BKF(A), 2BT9(C), 2C8S(A), 2CE2(X), 2CS7(A), 2D5M(A), 2DRT(A), 2DY0(A), 2EH3(A), 2F22(A), 2FB6(A), 2FJ8(A), 2FUP(A), 2GB4(B), 2GPI(A), 2GYQ(A), 2HEW(F), 2HW2(A), 2I8T(A), 2II2(A), 2IW1(A), 2J5Y(A), 2JE6(I), 2NLR(A), 2NSZ(A), 2O5G(B), 2OCT(A), 2OKT(A), 2OVG(A), 2P14(A), 2PA7(A), 2PND(A), 2PVB(A), 2QCP(X), 2QKV(A), 2QT1(A), 2R6J(A), 2RFF(A), 2UU8(A),

2V52(M), 2VB1(A), 2VK8(A), 2VXN(A), 2W3G(A), 2W91(A), 2WJ5(A), 2WQF(A), 2WWZ(C), 2X4L(A), 2XET(A), 2XOD(A), 2XU3(A), 1W7C(A), 1WLZ(A), 1WVQ(A), 1X8Q(A), 1XMT(A), 1Y93(A), 1YPY(A), 1Z6M(A), 1ZUU(A), 2AGK(A), 2ASB(A), 2BAY(A), 2BKX(A), 2BU3(A), 2C92(A), 2CG7(A), 2CVE(A), 2D5W(A), 2DRX(A), 2E3H(A), 2EHP(A), 2F23(A), 2FBA(A), 2FKK(A), 2FVY(A), 2GGC(A), 2GRR(B), 2GZ4(A), 2HHC(A), 2HWN(E), 2IA1(A), 2IJ2(A), 2IXM(A), 2J6B(A), 2JEK(A), 2NLS(A), 2NW8(A), 2O60(B), 2ODI(A), 2OLM(A), 2OVJ(A), 2P2S(A), 2PAG(A), 2PNE(A), 2PXX(A), 2QED(A), 2QL8(A), 2QXF(A), 2R6U(A), 2RFV(A), 2UV4(A), 2V6V(A), 2VBK(A), 2VLA(A), 2VXT(I), 2W40(A), 2WAG(A), 2WK1(A), 2WQK(B), 2WY4(A), 2X4W(B), 2XFR(A), 2XOL(A), 2XUV(A), 1WB4(A), 1WMH(A), 1WWI(A), 1X91(A), 1XPP(A), 1Y9L(A), 1YS1(X), 1Z6N(A), 1ZV1(B), 2AIB(A), 2ASK(A), 2BBR(A), 2BL8(A), 2BWR(A), 2CAR(A), 2CI1(A), 2CWS(A), 2D68(A), 2DS5(A), 2E3N(A), 2EHZ(A), 2F3Y(B), 2FCJ(A), 2FMA(A), 2FWH(A), 2GJ3(A), 2GS5(A), 2GZS(A), 2HIN(A), 2HX0(A), 2IA7(A), 2IMF(A), 2IYV(A), 2J73(B), 2JFR(A), 2NLV(A), 2NWF(A), 2O6N(A), 2ODK(A), 2OLN(A), 2OXG(Z), 2P4F(A), 2PBD(V), 2POF(A), 2PY5(A), 2QF4(A), 2QNG(A), 2QZC(A), 2R9F(A), 2RH2(A), 2UVK(A), 2V76(A), 2VC8(A), 2VNG(A), 2VY8(A), 2W47(A), 2WCW(A), 2WLV(A), 2WSD(A), 2WZB(A), 2X5N(A), 2XHF(B), 2XOM(A), 2XW6(A), 1WCW(A), 1WN2(A), 1WY3(A), 1X9I(A), 1XQO(A), 1YB3(A), 1YU0(A), 1Z72(A), 1ZZK(A), 2AKF(A), 2AXW(A), 2BCM(B), 2BLN(A), 2BZ1(A), 2CB8(A), 2CIO(B), 2CXA(A), 2DDR(A), 2DSK(A), 2E4T(A), 2END(A), 2F46(A), 2FCL(A), 2FP1(A), 2FYF(A), 2GJ4(A), 2GU9(A), 2H1V(A), 2HIY(A), 2HX5(A), 2IA8(A), 2IMH(A), 2IZR(A), 2J8K(A), 2JG0(A), 2NML(A), 2NWR(A), 2O7A(A), 2OFC(A), 2OML(A), 2OY9(A), 2P4H(X), 2PC1(A), 2PQ7(A), 2PYQ(A), 2QFA(C), 2QNL(A), 2R01(A), 2RA9(A), 2RHF(A), 2UY2(A), 2V7F(A), 2VCH(A), 2VOV(A), 2VZC(A), 2W50(A), 2WDC(A), 2WNF(A), 2WTP(A), 2WZO(A), 2X5O(A), 2XHG(A), 2XQQ(A), 2XWV(A), 2XXG(A), 2YH6(A), 2Z0J(A), 2ZDP(A), 2ZQ0(A), 3A0Y(A), 3A8G(B), 3AJ7(A), 3B0X(A), 3B9T(B), 3BM7(A), 3BUU(A), 3C8L(B), 3CI3(A), 3COV(A), 3D06(A), 3D7J(A), 3DLC(A), 3DSO(A), 3EF8(A), 3EQX(B), 3F43(A), 3FGV(A), 3FSS(A), 3G5S(A), 3GDM(A), 3GMX(B), 3GPI(A), 3H5J(A), 3HDX(A), 3HR6(A), 3I7M(A), 3IIS(M), 3ISX(A), 3IX3(A), 3JXO(A), 3KB9(A), 3KKF(A), 3KYJ(A), 3LAA(A), 3LKM(A), 3LW3(A), 3M12(A), 3MAB(A), 3MEA(A), 3MST(A), 3N01(A), 3NBC(A), 3NJN(A), 3NVS(A), 3O4P(A), 3OE3(A), 3OND(A), 3P0K(A), 3PIB(A), 3POJ(A), 3PUC(A), 3Q7R(A), 3QPA(A), 3QZX(A), 3R9F(A), 3RPD(A), 3S2J(A), 3S9X(A), 2XZ2(A),

2YHG(A), 2Z26(A), 2ZEX(B), 2ZS0(C), 3A16(A), 3A9S(A), 3AJD(A), 3B34(A), 3B9W(A), 3BMZ(A), 3BUX(B), 3C8Z(A), 3CIJ(A), 3CP7(A), 3D0J(A), 3D9N(A), 3DMG(A), 3DXL(A), 3EJV(A), 3ESS(A), 3F6Y(A), 3FGY(A), 3FTD(A), 3G5T(A), 3GE3(E), 3GNE(A), 3GRD(B), 3H6J(A), 3HF5(A), 3HS3(A), 3I94(A), 3IJL(A), 3IT3(A), 3IXL(A), 3JYO(A), 3KC2(A), 3KM5(A), 3KYZ(A), 3LAX(A), 3LLO(A), 3LWX(A), 3M3P(A), 3MAO(A), 3MIL(A), 3MT0(A), 3N08(A), 3NBM(A), 3NKE(A), 3NVW(B), 3O7B(A), 3OFG(A), 3ORU(A), 3P4H(A), 3PIU(A), 3PON(A), 3PVH(A), 3Q8J(A), 3QR7(A), 3R2Q(A), 3RJU(A), 3RPE(A), 3S2R(A), 3SBM(A), 2Y5P(D), 2YIM(A), 2Z51(A), 2ZFD(B), 2ZUX(B), 3A1H(A), 3ABN(A), 3AKS(A), 3B4Q(A), 3BA3(A), 3BO6(A), 3BVX(A), 3C9A(A), 3CIM(A), 3CT5(A), 3D1P(A), 3D9X(A), 3DNJ(A), 3DXY(A), 3EKI(A), 3EUN(A), 3F7E(A), 3FIL(A), 3FWK(A), 3G7R(A), 3GG7(A), 3GNL(A), 3GWI(A), 3H74(A), 3HKW(A), 3HWU(A), 3IB5(A), 3IKW(A), 3ITF(A), 3JQ0(A), 3K05(A), 3KE7(B), 3KMH(A), 3KZ5(E), 3LB2(A), 3LLU(A), 3LX3(A), 3M5Q(A), 3MBR(X), 3MJF(A), 3MVC(A), 3N0R(A), 3ND1(A), 3NNB(A), 3NYC(A), 3O9Z(A), 3OG2(A), 3OV5(A), 3PB6(X), 3PIW(A), 3POW(A), 3PVI(A), 3QB8(A), 3QU3(A), 3R41(A), 3RKG(A), 3RQ9(A), 3S44(A), 3SC7(X), 2Y78(A), 2YKZ(A), 2Z5W(A), 2ZHJ(A), 2ZW2(A), 3A2Z(A), 3ACH(A), 3AMR(A), 3B5M(A), 3BB0(A), 3BOE(A), 3BWH(A), 3C9U(A), 3CJM(A), 3CT6(A), 3D2Q(A), 3DB7(A), 3DO8(A), 3E0X(A), 3ELF(A), 3EUR(A), 3F8X(D), 3FJU(B), 3FYM(A), 3G89(A), 3GIU(A), 3GNZ(P), 3GZB(F), 3H75(A), 3HLX(A), 3HX8(B), 3IB7(A), 3IMK(A), 3ITQ(A), 3JRV(C), 3K0B(A), 3KEV(A), 3KOR(A), 3L1W(A), 3LD7(A), 3LM3(A), 3LYD(A), 3M66(A), 3MC3(A), 3MJV(A), 3MVS(A), 3N10(A), 3NDH(A), 3NO0(A), 3NYT(A), 3OAJ(A), 3OGN(A), 3OV9(A), 3PD7(A), 3PJP(A), 3PP2(A), 3Q1C(A), 3QC7(A), 3QU5(A), 3R4Z(A), 3RL5(A), 3RRI(A), 3S4E(A), 3SCY(A), 2Y7E(A), 2YN0(A), 2Z6O(A), 2ZK9(X), 2ZWS(A), 3A35(A), 3ACX(A), 3AYJ(A), 3B5N(C), 3BED(A), 3BON(A), 3BWZ(A), 3CA7(A), 3CJS(B), 3CTZ(A), 3D3B(J), 3DHA(A), 3DOU(A), 3E4G(A), 3EN0(A), 3EYE(A), 3FCN(A), 3FKE(A), 3FYN(A), 3G91(A), 3GIW(A), 3GOC(B), 3GZR(B), 3H7H(B), 3HLZ(A), 3HYN(A), 3IE4(A), 3IP0(A), 3IUO(A), 3JS8(A), 3K1U(A), 3KFF(A), 3KPE(B), 3L46(A), 3LFK(A), 3LMZ(A), 3LYE(A), 3M6Z(A), 3MCW(B), 3MMH(A), 3MWX(A), 3N17(A), 3NEU(A), 3NO7(A), 3NZL(A), 3OBL(A), 3OHE(A), 3OX7(P), 3PE6(A), 3PL8(A), 3PP4(P), 3Q1X(A), 3QGU(B), 3QVP(A), 3R5T(A), 3RLG(A), 3RT2(A), 3S5M(A), 3SEE(A), 2Y8Y(A), 2YOG(A), 2Z6R(A), 2ZNR(A), 3A02(A), 3A57(A), 3ADM(A), 3AZD(A), 3B5O(A), 3BEX(A), 3BQP(A), 3BY8(A), 3CAY(A), 3CKM(A), 3CUZ(A),

3D40(A), 3DJE(B), 3DQY(A), 3E7R(L), 3EO6(B), 3F0D(A), 3FDE(A), 3FMY(A), 3G0K(A), 3GA4(A), 3GJY(A), 3GOE(A), 3H0N(A), 3H7I(A), 3HM4(B), 3HZP(A), 3IE7(A), 3IPJ(A), 3IUW(B), 3JTZ(A), 3K1Z(A), 3KGK(A), 3KTP(B), 3L51(B), 3LHC(A), 3LO8(A), 3LYH(B), 3M73(A), 3MD7(A), 3MN5(S), 3MWZ(A), 3N2W(A), 3NFT(A), 3NOH(A), 3NZM(A), 3OBQ(A), 3OM0(A), 3OXP(A), 3PES(A), 3PLW(A), 3PP5(A), 3Q46(A), 3QHB(A), 3QX1(A), 3R6D(A), 3RO3(B), 3RWN(A), 3S6E(A), 3SGG(A), 2YC3(A), 2YV9(A), 2Z72(A), 2ZOU(A), 3A09(A), 3A5F(A), 3AGN(A), 3B0F(B), 3B64(A), 3BF7(A), 3BRC(A), 3C2U(A), 3CBZ(A), 3CL6(A), 3CWR(B), 3D4E(A), 3DK9(A), 3DS4(A), 3E8O(B), 3EOI(A), 3F14(A), 3FEG(A), 3FRH(A), 3G16(A), 3GA7(A), 3GKJ(A), 3GOH(A), 3H3L(B), 3H87(C), 3HO6(A), 3I10(A), 3IEZ(A), 3IPN(A), 3IV4(A), 3JU4(A), 3K5J(A), 3KGY(B), 3KU3(B), 3L81(A), 3LHI(A), 3LQB(A), 3LYP(A), 3M7A(B), 3MDQ(A), 3MQD(A), 3MXN(B), 3N3M(A), 3NGG(A), 3NPD(A), 3O12(A), 3OCJ(A), 3OMD(A), 3OYV(A), 3PF6(A), 3PMS(A), 3PPL(A), 3Q4O(A), 3QL9(A), 3QZB(A), 3R72(A), 3ROB(A), 3RX9(A), 3S6F(A), 3SHG(B), 2YFO(A), 2YVT(A), 2Z98(A), 2ZPM(A), 3A0M(A), 3A6R(A), 3AH9(A), 3B0G(A), 3B6E(A), 3BGU(A), 3BS4(A), 3C70(A), 3CCD(A), 3CLM(A), 3CXN(A), 3D59(A), 3DKM(A), 3DSB(A), 3E8T(A), 3EOJ(A), 3F1L(A), 3FG9(A), 3FSA(A), 3G21(A), 3GAE(A), 3GKR(A), 3GP2(B), 3H4O(A), 3H8G(F), 3HP7(A), 3I2K(A), 3IFE(A), 3IQU(A), 3IVV(A), 3JUD(A), 3K67(A), 3KH1(B), 3KWE(A), 3L8W(A), 3LHN(A), 3LT7(A), 3M0M(A), 3M8J(A), 3MDU(A), 3MQZ(A), 3MXZ(A), 3N6Y(A), 3NIR(A), 3NR5(A), 3O2R(A), 3OCU(A), 3OMY(A), 3P02(A), 3PFE(A), 3PN3(B), 3PSM(A), 3Q64(A), 3QOO(A), 3QZM(A), 3R87(A), 3ROF(A), 3RZN(A), 3S83(A), 3SIG(A), 2YH5(A), 2YZY(A), 2ZCM(A), 2ZPT(X), 3A0S(A), 3A72(A), 3AIA(A), 3B0S(A), 3B79(A), 3BHW(A), 3BT5(A), 3C8C(A), 3CHM(A), 3CMB(D), 3CZX(A), 3D5P(A), 3DKR(A), 3DSK(A), 3EA6(A), 3EPR(A), 3F2Z(A), 3FGH(A), 3FSO(A), 3G36(C), 3GBW(A), 3GMG(A), 3GP6(A), 3H4T(A), 3H9M(A), 3HPW(C), 3I4G(A), 3IFN(P), 3IR4(A), 3IWF(A), 3JUM(A), 3K6Y(A), 3KIZ(A), 3KWR(A), 3L9A(X), 3LJM(A), 3LTI(A), 3M0Z(A), 3M9Q(A), 3ME7(A), 3MR0(A), 3MYX(A), 3N6Z(A), 3NJ2(A), 3NUF(A), 3O2T(A), 3ODV(A), 3ON9(A), 3P0B(A), 3PFG(A), 3POD(A), 3PT5(A), 3Q6B(A), 3QP4(A), 3QZR(A), 3R8J(A), 3RPC(A), 3S0A(A), 3S8S(A), 3SJM(A), 3SK2(A), 3SU6(A), 3T47(A), 3TG0(A), 3TS3(A), 3U65(B), 3UFF(A), 3UR8(A), 3V68(A), 3VL1(A), 3VUP(A), 3W0O(A), 3WH1(A), 3WQB(B), 3WWX(A), 3ZFP(A), 3ZRX(A), 3ZY7(A), 4A37(A), 4AC1(X), 4ANN(A), 4AXY(A), 4B6G(A), 4BGC(A), 4BRC(A), 4C5K(A), 4CFI(A), 4CNN(A), 4D0Q(A),

4DM5(A), 4DQJ(B), 4E2X(A), 4EBG(A), 4EQ8(A), 4ESA(B), 4EZI(A), 4FCH(B), 4FZL(A), 4G78(A), 4GHN(A), 4GUC(A), 4H4D(A), 4HC9(A), 4HJI(A), 4HTG(A), 4I6R(A), 4IFA(A), 4IUS(A), 4JB7(A), 4JHT(A), 4JXR(A), 4KH8(A), 4L0N(A), 4LD1(A), 4LLD(B), 4LZX(B), 4M91(A), 4MF5(A), 4MUV(A), 4N1I(A), 4NES(A), 4NPD(A), 4O6U(A), 4OM8(A), 3SK7(A), 3SUK(A), 3T4L(A), 3TG2(A), 3TUT(A), 3U6G(A), 3UI4(A), 3URR(A), 3V7N(A), 3VLA(A), 3VVI(A), 3W19(D), 3WIW(A), 3WQC(A), 3WZ3(A), 3ZHI(A), 3ZSJ(A), 3ZYP(A), 4A4J(A), 4ACJ(A), 4AQO(A), 4AY0(A), 4B89(A), 4BJ0(A), 4BT7(A), 4C6A(A), 4CFQ(Q), 4CO8(A), 4D6G(A), 4DMI(A), 4DRI(B), 4E3Y(A), 4EBJ(A), 4EQ9(A), 4ESM(A), 4F06(A), 4FFL(A), 4FZP(A), 4G7X(B), 4GJZ(A), 4GVF(B), 4H4N(A), 4HCJ(A), 4HLY(A), 4HVK(A), 4I6X(A), 4IGI(A), 4IX7(A), 4JBD(A), 4JJA(A), 4JZ5(A), 4KL0(A), 4L2H(A), 4LDV(A), 4LPQ(A), 4M0W(A), 4M9K(A), 4MFI(A), 4MXT(A), 4N2K(A), 4NG0(A), 4NSV(A), 4OA3(A), 4ONR(A), 3SNO(A), 3SXM(A), 3T7H(A), 3TG7(A), 3TVJ(A), 3U7Z(A), 3UID(A), 3UXJ(A), 3VEJ(A), 3VMK(A), 3VVV(A), 3W42(A), 3WJT(A), 3WS7(A), 3X0I(A), 3ZJA(A), 3ZSU(A), 3ZZO(A), 4A4Y(A), 4AE7(A), 4ARU(A), 4AYO(A), 4B8X(A), 4BJI(A), 4BTV(A), 4CA1(A), 4CGS(A), 4COG(A), 4D7J(A), 4DMT(A), 4DT5(A), 4E40(A), 4EE6(A), 4EQA(C), 4ESW(A), 4F1V(A), 4FK9(A), 4G0X(A), 4G9E(A), 4GMQ(A), 4GVQ(A), 4H5I(A), 4HCS(A), 4HMS(A), 4HWM(A), 4I6Y(A), 4IIK(A), 4J42(A), 4JCC(A), 4JK8(A), 4JZZ(A), 4KM6(A), 4L2I(B), 4LEB(A), 4LRT(A), 4M1G(A), 4M9V(F), 4MIY(A), 4MYD(A), 4N2P(D), 4NI6(A), 4NUT(B), 4OCV(A), 4OPC(A), 3SO6(A), 3SY1(A), 3T7L(A), 3TJM(A), 3TVT(B), 3U97(A), 3UJC(A), 3V0D(A), 3VEN(A), 3VMN(A), 3VWC(A), 3W7T(A), 3WKG(A), 3WUP(A), 3X0T(A), 3ZN4(A), 3ZUC(A), 3ZZP(A), 4A56(A), 4AF8(A), 4AT0(A), 4AZ6(A), 4B9G(A), 4BK7(A), 4BVX(A), 4CAY(C), 4CHI(A), 4CS4(A), 4D8B(A), 4DMV(A), 4DUQ(A), 4E4R(A), 4EFP(A), 4EQB(A), 4EU9(A), 4F2E(A), 4FN7(A), 4G1Q(B), 4G9S(B), 4GMU(A), 4GWB(A), 4H6C(I), 4HDD(A), 4HNO(A), 4HY4(A), 4I71(A), 4IIL(A), 4J5R(A), 4JDU(A), 4JM1(A), 4K12(B), 4KQD(A), 4L57(A), 4LF0(A), 4LRU(A), 4M1U(A), 4MAI(A), 4MJD(A), 4MYK(A), 4N30(A), 4NKP(A), 4NXY(A), 4OD6(A), 4OQ9(A), 3SOJ(A), 3SZ3(A), 3T90(A), 3TM8(A), 3TYS(A), 3U9R(B), 3UJI(P), 3V1A(A), 3VGI(A), 3VOR(A), 3VWN(X), 3W7Y(B), 3WMT(B), 3WUR(A), 3X2M(A), 3ZN6(A), 3ZUZ(A), 3ZZS(A), 4A6Q(A), 4AFF(A), 4ATE(A), 4B0Z(A), 4BB9(A), 4BN4(A), 4BY8(A), 4CC2(A), 4CI9(A), 4CSR(A), 4DB5(A), 4DN7(A), 4DWR(A), 4E6F(A), 4EGU(A), 4EQP(A), 4EUN(A), 4F2L(A), 4FNV(A), 4G3B(A), 4GA2(A), 4GNR(A), 4GXW(A), 4H6Q(A), 4HE6(A),

4HQZ(A), 4I0W(A), 4I84(A), 4IKD(A), 4J6O(A), 4JEM(A), 4JMP(A), 4K7B(A), 4KQI(A), 4L5E(A), 4LGJ(A), 4LTT(A), 4M1X(A), 4MAK(A), 4MNC(A), 4MYZ(A), 4N7F(A), 4NLM(A), 4NYQ(A), 4OE9(A), 4OQP(A), 3SOV(A), 3SZV(A), 3T92(A), 3TOS(A), 3U2U(A), 3U9W(A), 3ULJ(A), 3V1N(A), 3VGL(A), 3VQJ(A), 3VZ9(D), 3WA2(X), 3WMV(B), 3WV7(A), 3X34(A), 3ZNV(A), 3ZVS(A), 3ZZY(A), 4A6R(A), 4AFM(A), 4AU1(A), 4B1M(B), 4BEU(A), 4BND(A), 4BYZ(A), 4CD5(A), 4CIM(P), 4CUA(A), 4DI9(A), 4DO4(A), 4DYQ(A), 4E74(A), 4EIC(A), 4EQS(A), 4EUO(A), 4F54(A), 4FP5(D), 4G3O(A), 4GB5(A), 4GOF(A), 4GYX(A), 4H7W(A), 4HFS(A), 4HRO(A), 4I1K(A), 4I8H(A), 4IL7(A), 4J7N(A), 4JF8(A), 4JN7(A), 4K82(A), 4KQP(A), 4L8P(A), 4LGY(A), 4LUA(A), 4M2M(A), 4MAQ(A), 4MNO(A), 4MZC(A), 4N8C(X), 4NN2(A), 4NZK(A), 4OH7(A), 4OU0(A), 3SQZ(A), 3SZY(A), 3TC8(A), 3TOW(A), 3U3L(C), 3UB6(A), 3ULT(A), 3V46(A), 3VII(A), 3VRD(B), 3W06(A), 3WDC(A), 3WN7(B), 3WVA(A), 3ZBD(A), 3ZOJ(A), 3ZW5(A), 4A02(A), 4A7U(A), 4AK2(A), 4AVS(E), 4B1Y(M), 4BFH(A), 4BOU(A), 4C08(A), 4CD8(A), 4CJ0(B), 4CV7(A), 4DJA(A), 4DPZ(X), 4DZI(A), 4EA9(A), 4EKF(A), 4ERC(A), 4EVU(A), 4F87(A), 4FR9(A), 4G4K(A), 4GC3(A), 4GS3(A), 4GZC(A), 4H8E(A), 4HGU(A), 4HS1(A), 4I3G(B), 4IAU(A), 4INW(A), 4J8C(A), 4JG2(A), 4JNU(A), 4KBX(A), 4KT3(B), 4L9N(A), 4LHS(A), 4LUP(A), 4M51(A), 4MAX(A), 4MTM(A), 4MZJ(A), 4NBP(A), 4NNO(A), 4O06(A), 4OHJ(A), 4OY5(A), 3SRI(B), 3T2C(A), 3TDU(D), 3TPD(A), 3U3Z(A), 3UC7(A), 3UP3(A), 3V4K(A), 3VJ9(A), 3VSV(B), 3W07(A), 3WDN(A), 3WN8(A), 3WWC(A), 3ZBO(B), 3ZOQ(B), 3ZXC(A), 4A29(A), 4A9V(A), 4AL0(A), 4AW7(A), 4B4D(A), 4BFO(A), 4BPF(A), 4C1A(A), 4CDP(A), 4CK4(A), 4CZ5(A), 4DJC(B), 4DQ9(A), 4E15(A), 4EAD(A), 4EMN(A), 4ES1(A), 4EX6(A), 4F98(A), 4FS7(A), 4G54(A), 4GEI(A), 4GT8(A), 4H27(A), 4HBQ(A), 4HHR(A), 4HS2(A), 4I4O(A), 4IC4(A), 4IPU(A), 4J8S(A), 4JGI(A), 4JTM(A), 4KDW(A), 4KU0(D), 4L9P(B), 4LIX(A), 4LX2(B), 4M7T(A), 4MC3(A), 4MTU(A), 4N03(A), 4NDO(A), 4NOA(A), 4O0A(A), 4OI3(A), 4P0T(A), 3SS7(X), 3T3L(A), 3TEU(A), 3TQ2(A), 3U5S(A), 3UFE(A), 3UPL(A), 3V5C(A), 3VK5(A), 3VUB(A), 3W0K(A), 3WGX(C), 3WND(A), 3WWL(A), 3ZDB(A), 3ZR8(X), 3ZXK(A), 4A2V(A), 4AAN(A), 4ALZ(A), 4AXO(A), 4B5O(A), 4BGB(A), 4BQN(A), 4C2V(C), 4CE8(C), 4CNG(A), 4D0P(A), 4DK2(A), 4DQA(A), 4E29(A), 4EAE(A), 4EP4(A), 4ES8(A), 4EZG(A), 4FBJ(A), 4FTF(A), 4G6T(B), 4GEK(A), 4GT9(A), 4H3U(A), 4HBZ(A), 4HI8(B), 4HST(B), 4I66(A), 4IEJ(A), 4IUM(A), 4J9Y(B), 4JGL(A), 4JVO(A), 4KEF(A), 4KV7(A), 4LA2(A), 4LKU(A), 4LYP(A), 4M8A(A), 4ME2(A),

4MUQ(A), 4N13(A), 4NDS(A), 4NOH(A), 4O0C(A), 4OJX(A), 4P3H(A), 4P3V(A), 4PEY(A), 4PLZ(A), 4PVK(A), 4Q2L(A), 4QA8(A), 4QI3(B), 4QTC(A), 4R3N(A), 4RBX(A), 4RJZ(A), 4RU3(A), 4RYO(A), 4TR6(A), 4U5R(A), 4UFQ(A), 4V0W(B), 4W6Y(A), 4WBJ(A), 4WHS(D), 4WPG(A), 4WY9(A), 4X9X(A), 4XED(A), 4XPX(A), 4Y1W(A), 4YCB(A), 4YMY(A), 4YTW(D), 4Z0W(B), 4ZBG(A), 4ZIL(A), 4ZRX(A), 5A0Y(C), 5A7V(A), 5AGR(A), 5AOG(A), 5B1A(Y), 5B8D(A), 5BTY(A), 5C3F(B), 5CGQ(B), 5CR4(A), 5CWL(A), 5DHD(A), 5DU9(A), 5E9P(A), 5EMI(A), 5EWY(A), 5FBF(A), 5FQE(A), 5G51(A), 5GRQ(C), 5H0Q(A), 5HC0(A), 5HQH(A), 5I39(A), 5IGI(A), 5IPY(A), 5J4F(A), 5JDA(A), 5JO8(A), 5JVI(E), 5KAR(A), 4P40(A), 4PF3(A), 4PNO(A), 4PW0(A), 4Q2S(A), 4QAS(A), 4QKD(A), 4QXB(C), 4R5R(A), 4RD4(A), 4RK4(A), 4RUQ(B), 4RZ9(A), 4TSD(B), 4U98(A), 4UHT(A), 4V1G(A), 4W78(H), 4WBY(A), 4WIQ(A), 4WPK(A), 4WZX(E), 4X9Z(A), 4XEM(A), 4XQ7(A), 4Y2F(A), 4YDR(B), 4YNH(A), 4YUC(A), 4Z1R(A), 4ZBH(A), 4ZJH(A), 4ZV0(B), 5A1I(A), 5A8C(A), 5AHK(A), 5AOT(A), 5B1R(A), 5BJX(A), 5BXR(B), 5C5G(A), 5CIY(A), 5CSD(A), 5D2K(A), 5DIC(A), 5DVI(A), 5EC6(A), 5EMX(A), 5EXH(C), 5FEB(A), 5FS8(A), 5G5C(A), 5GS7(A), 5H1N(A), 5HDK(A), 5HRA(A), 5I45(A), 5IHF(A), 5ISV(A), 5J4L(A), 5JDK(A), 5JOV(A), 5JXM(A), 5KDS(A), 4P5E(A), 4PH2(A), 4PP4(A), 4PWO(A), 4Q3K(A), 4QBO(A), 4QLP(B), 4QXL(A), 4R6H(A), 4RDB(A), 4RLC(A), 4RUW(A), 4S1P(A), 4TTN(A), 4U9H(S), 4UJ7(A), 4V1J(A), 4W79(A), 4WCK(A), 4WJI(A), 4WPY(A), 4X1Z(B), 4XB4(A), 4XEZ(A), 4XQC(A), 4Y6W(A), 4YE7(A), 4YNX(A), 4YWA(A), 4Z39(A), 4ZBO(D), 4ZJU(A), 4ZV5(A), 5A1Q(A), 5A8J(A), 5AIG(A), 5AOZ(A), 5B4B(B), 5BK9(B), 5BY5(A), 5C5Z(A), 5CKL(A), 5CTD(C), 5D4V(A), 5DJH(A), 5DWA(B), 5ECK(A), 5EP6(B), 5EZU(A), 5FEW(A), 5FSV(A), 5GGB(A), 5GTQ(A), 5H3J(B), 5HEE(A), 5HSG(A), 5I5B(A), 5IHW(A), 5ITM(A), 5J4O(A), 5JE2(B), 5JPH(A), 5K2L(A), 5KF9(A), 4P5N(A), 4PH8(A), 4PQD(A), 4PWW(A), 4Q4G(X), 4QC6(A), 4QM6(A), 4QXV(A), 4R75(A), 4REI(A), 4RLZ(A), 4RVQ(A), 4S28(A), 4TTW(A), 4U9O(A), 4UQW(A), 4V1K(A), 4W7L(A), 4WDC(A), 4WJT(A), 4WQK(A), 4X2R(A), 4XBA(B), 4XFK(A), 4XTB(A), 4Y7L(A), 4YEC(B), 4YOR(A), 4YWK(A), 4Z3G(A), 4ZC3(A), 4ZLD(A), 4ZVC(A), 5A3A(A), 5A95(B), 5AIM(A), 5APG(A), 5B4Z(A), 5BMN(A), 5BY8(B), 5C8Z(A), 5CL8(A), 5CTM(A), 5D5Y(B), 5DKX(A), 5DXL(A), 5EDF(A), 5EQ0(A), 5F2K(A), 5FIS(A), 5FU5(A), 5GGN(A), 5GTU(B), 5H3V(A), 5HHE(D), 5HTL(A), 5I5N(A), 5II6(A), 5IWH(A), 5J6Y(A), 5JEL(B), 5JQF(A), 5K4B(A), 5KHT(A), 4P7O(A), 4PHJ(A), 4PQH(A), 4PXE(A), 4Q4W(4), 4QDJ(A), 4QOS(A),

4QY7(A), 4R78(A), 4REK(A), 4RP3(A), 4RWC(A), 4S39(A), 4TVV(A), 4UA6(A), 4UQX(A), 4V2K(A), 4W7W(A), 4WE2(A), 4WKA(A), 4WSF(A), 4X5P(A), 4XD1(A), 4XFM(A), 4XUW(A), 4Y88(A), 4YEP(A), 4YQD(A), 4YX1(A), 4Z47(A), 4ZCE(B), 4ZMK(A), 4ZVF(A), 5A61(A), 5A99(A), 5AJG(A), 5AQ0(A), 5B5I(A), 5BMT(A), 5BYK(A), 5C98(A), 5COF(A), 5CTV(A), 5D66(A), 5DLE(A), 5DZE(A), 5EHI(A), 5EQ7(A), 5F47(A), 5FJD(A), 5FVN(D), 5GI7(A), 5GV0(A), 5H6X(A), 5HHJ(A), 5HTX(A), 5I90(A), 5II8(A), 5IXB(A), 5J90(A), 5JGK(A), 5JRT(A), 5K6D(A), 5KI9(A), 4P7X(A), 4PHR(A), 4PS6(A), 4PXY(A), 4Q53(A), 4QEK(A), 4QP5(A), 4R03(A), 4R81(A), 4REO(A), 4RPM(A), 4RWU(A), 4TKC(A), 4TXR(C), 4UAB(A), 4UQZ(B), 4V33(A), 4W8B(A), 4WEE(A), 4WLH(A), 4WU0(A), 4X7G(A), 4XDQ(A), 4XIN(A), 4XXF(A), 4Y9I(A), 4YFU(A), 4YSI(A), 4YYC(A), 4Z7X(A), 4ZEY(A), 4ZOX(B), 4ZW9(A), 5A62(A), 5AE0(A), 5AL6(A), 5AZB(A), 5B6C(A), 5BOB(A), 5C12(A), 5CDK(A), 5COW(A), 5CUO(A), 5D8V(A), 5DM2(A), 5E1W(A), 5EJ8(A), 5EU0(B), 5F5N(A), 5FJL(A), 5FYD(A), 5GJI(A), 5GV8(A), 5H9N(A), 5HJ1(A), 5HUB(A), 5I95(A), 5IK4(A), 5IXH(A), 5JAZ(A), 5JH8(A), 5JRY(A), 5K86(A), 5KKO(A), 4P82(A), 4PI8(A), 4PSF(A), 4PZ0(A), 4Q7O(A), 4QGO(A), 4QPN(A), 4R16(A), 4R9P(A), 4RFU(A), 4RPT(A), 4RWW(A), 4TMX(A), 4TYZ(A), 4UDX(X), 4UU3(B), 4V3I(A), 4W8H(A), 4WF5(A), 4WN5(A), 4WUI(A), 4X84(A), 4XDU(A), 4XJ5(A), 4XXL(A), 4Y9V(A), 4YI8(A), 4YSL(A), 4YZ0(A), 4Z80(C), 4ZGF(A), 4ZPC(A), 4ZZ1(A), 5A67(A), 5AFY(L), 5AN5(J), 5AZW(A), 5B78(B), 5BOW(A), 5C17(A), 5CEC(A), 5COY(A), 5CVW(A), 5DBL(A), 5DMA(A), 5E4G(A), 5EL3(A), 5EW0(A), 5F6R(A), 5FLW(A), 5FYP(A), 5GNF(A), 5GWN(A), 5HB6(A), 5HJ9(A), 5HWA(A), 5IDB(A), 5IMA(A), 5J1N(A), 5JBN(A), 5JIC(A), 5JS4(A), 5K87(A), 5KLA(A), 4PDN(A), 4PIO(A), 4PSR(B), 4PZ3(A), 4Q7Q(A), 4QHQ(A), 4QPW(A), 4R1J(A), 4RAX(A), 4RGD(A), 4RRI(A), 4RXV(A), 4TPV(A), 4U0O(B), 4UE0(A), 4UYR(A), 4V4M(E), 4W8P(B), 4WFO(A), 4WND(B), 4WWF(A), 4X9R(A), 4XDX(A), 4XMR(A), 4XXX(A), 4Y9W(A), 4YKI(A), 4YTD(A), 4YZZ(A), 4Z9H(A), 4ZHB(A), 4ZQX(A), 5A0L(B), 5A6M(A), 5AGD(B), 5ANP(A), 5AZX(B), 5B7H(A), 5BR4(A), 5C2U(A), 5CEG(B), 5COZ(A), 5CWG(A), 5DCU(A), 5DMD(A), 5E5Y(A), 5EL9(A), 5EWO(A), 5FAF(A), 5FMU(A), 5G38(A), 5GNG(A), 5GZ3(A), 5HB7(A), 5HNV(A), 5HWK(A), 5IDH(A), 5INB(B), 5J1S(B), 5JBX(A), 5JIG(A), 5JUG(A), 5K8J(A), 5KLE(A), 4PDY(A), 4PJ2(A), 4PSS(A), 4Q29(A), 4Q98(A), 4QHW(A), 4QRN(A), 4R2X(C), 4RAY(A), 4RJW(A), 4RTH(A), 4RY1(A), 4TQX(A), 4U5H(A), 4UE8(B), 4UZG(A), 4W64(A), 4W9Z(A), 4WH9(A), 4WP9(A), 4WY4(D),

4X9T(A), 4XDZ(A), 4XOT(A), 4XZF(A), 4YAA(A), 4YL8(B), 4YTK(A), 4Z0G(A), 4ZAV(A), 4ZHW(A), 4ZR8(A), 5A0N(A), 5A71(A), 5AGI(A), 5ANV(A), 5B08(A), 5B7Y(A), 5BS1(A), 5C33(B), 5CGO(A), 5CPH(A), 5CWH(A), 5DGJ(A), 5DP2(A), 5E7H(A), 5ELB(C), 5EWU(A), 5FAV(B), 5FPZ(A), 5G3Y(A), 5GQI(A), 5H0M(A), 5HBP(A), 5HOE(A), 5I0Y(A), 5IG6(A), 5IO9(A), 5J3T(C), 5JCA(S), 5JJ2(A), 5JUH(A), 5K91(A), 5KNH(I), 5KO5(A), 5L0R(B), 5LB7(A), 5LND(A), 5LUS(A), 5M0Y(B), 5M5Z(A), 5MFO(A), 5MUL(A), 5N86(A), 5NMN(A), 5NW3(A), 5O45(A), 5OBY(A), 5OLR(C), 5Q22(A), 5RKZ(A), 5T5L(A), 5TLE(A), 5U5T(C), 5ULB(A), 5UXM(A), 5V6J(A), 5VNY(A), 5W8Q(A), 5WLJ(B), 5X5M(A), 5XBI(A), 5XM5(B), 5Y9X(A), 5YGB(A), 5YO6(A), 5YZP(A), 5ZHO(A), 5ZX9(A), 6A9S(A), 6AT6(A), 6B9H(B), 6BSC(B), 6C29(B), 6CDX(B), 6D0H(B), 6DGG(A), 6E0O(A), 6E68(A), 6EFN(A), 6EQE(A), 6EXM(A), 6FBQ(A), 6FIH(A), 6FME(B), 6FUC(A), 6G49(A), 6GCV(A), 6GIT(A), 6GSZ(A), 6H0C(A), 6H9U(B), 6HHM(A), 6HS0(A), 6HZR(A), 6I5R(A), 6IJE(B), 6J4D(A), 5KP7(B), 5L0V(B), 5LBD(A), 5LP9(A), 5LW3(A), 5M10(A), 5M72(B), 5MGW(A), 5MX9(A), 5N8A(X), 5NOA(A), 5NWP(A), 5O58(A), 5OD4(A), 5OMT(A), 5QHH(A), 5SUI(A), 5T7A(A), 5TOQ(A), 5U81(A), 5UMR(A), 5UZG(B), 5V8S(A), 5VX5(A), 5W98(A), 5WN9(A), 5X5V(A), 5XCO(B), 5XVE(A), 5Y9Z(A), 5YH4(A), 5YP7(A), 5Z0D(B), 5ZKE(B), 5ZZA(P), 6AC5(A), 6ATW(A), 6B9X(E), 6BSU(A), 6C3C(A), 6CHX(A), 6D4K(A), 6DGM(B), 6E1F(B), 6E6O(A), 6EHI(A), 6ER1(A), 6EXX(A), 6FC0(B), 6FIY(B), 6FNU(A), 6FVI(A), 6G4J(A), 6GDJ(A), 6GKX(A), 6GV8(A), 6H10(A), 6HA4(A), 6HHN(A), 6HSA(A), 6I03(A), 6I6M(A), 6IQC(A), 6J4K(B), 5KTN(A), 5L37(C), 5LEO(A), 5LPA(A), 5LXE(A), 5M17(A), 5M7Y(A), 5MJR(A), 5MY5(A), 5NAK(A), 5NQO(A), 5NZG(A), 5O5T(A), 5OE3(A), 5ONK(A), 5QI0(A), 5SV2(A), 5T8C(A), 5TPI(A), 5UAM(A), 5UOU(A), 5V01(A), 5VBD(A), 5VXV(A), 5WD9(A), 5WQJ(A), 5X7L(A), 5XDC(A), 5XVJ(A), 5YA6(A), 5YHR(A), 5YQJ(A), 5Z3E(A), 5ZO3(A), 6A02(A), 6AJP(A), 6AVX(A), 6BA9(A), 6BTD(A), 6C4Q(A), 6CKA(B), 6D9N(A), 6DKQ(A), 6E1Z(A), 6E6Q(B), 6EIO(A), 6ER4(B), 6EY1(A), 6FC1(B), 6FJN(A), 6FOP(A), 6FXA(A), 6G6K(A), 6GDX(B), 6GM5(A), 6GVD(A), 6H20(A), 6HAV(A), 6HIU(B), 6HSJ(A), 6I05(A), 6I8Y(A), 6ITA(A), 6J4P(B), 5KVB(A), 5L74(A), 5LHM(A), 5LQ5(A), 5LXZ(B), 5M1M(A), 5M97(B), 5MK9(A), 5MY7(A), 5NCJ(A), 5NR4(B), 5NZO(B), 5O63(A), 5OF1(A), 5OPZ(A), 5QIV(A), 5SV5(A), 5TAB(A), 5TQI(A), 5UBA(A), 5UQ6(A), 5V1V(D), 5VCM(A), 5W0G(A), 5WEC(A), 5WRI(A), 5X89(A), 5XDH(C), 5XVT(A), 5YAY(B), 5YIU(A), 5YRV(C), 5Z42(A), 5ZOH(A), 6A0A(A),

6AKK(A), 6AZM(F), 6BCB(A), 6BUM(D), 6C52(D), 6CNW(A), 6DCE(A), 6DNM(A), 6E3A(A), 6E6U(B), 6EKL(B), 6ETL(A), 6EYG(A), 6FDG(A), 6FLF(A), 6FPQ(A), 6FXD(A), 6G7N(A), 6GEH(A), 6GN5(A), 6GVK(B), 6H24(A), 6HBB(A), 6HK9(B), 6HTO(A), 6I0I(A), 6I9A(A), 6ITG(A), 6J6P(A), 5KVS(B), 5L77(A), 5LHW(A), 5LQ6(A), 5LY3(A), 5M1P(A), 5MAO(A), 5ML3(B), 5MYC(P), 5NFM(A), 5NRH(B), 5O0S(A), 5O6H(A), 5OHQ(A), 5OQ3(A), 5QOQ(A), 5SVY(A), 5TCB(A), 5TRQ(A), 5UE1(A), 5UQS(C), 5V1Y(B), 5VEO(A), 5W2F(A), 5WFY(A), 5WSF(A), 5X9I(A), 5XEV(A), 5XZ7(A), 5YCA(C), 5YJ6(A), 5YSI(A), 5Z51(A), 5ZQY(A), 6A0C(A), 6AM3(X), 6B1K(A), 6BCD(B), 6BW9(B), 6C8C(B), 6COJ(B), 6DCJ(A), 6DNO(B), 6E3I(B), 6E7E(A), 6EKZ(A), 6EUW(A), 6F0W(S), 6FF1(A), 6FLK(A), 6FQ1(A), 6FYJ(A), 6G8Y(A), 6GEU(A), 6GP3(A), 6GX2(A), 6H40(A), 6HCW(A), 6HN1(A), 6HUR(A), 6I18(A), 6IBE(A), 6IUX(A), 6J93(A), 5KXH(B), 5L87(A), 5LHX(A), 5LS4(A), 5LY8(A), 5M29(A), 5MAW(E), 5MPR(A), 5N0O(A), 5NGN(A), 5NRM(A), 5O0U(A), 5O75(A), 5OK6(A), 5OUO(A), 5QS9(A), 5SY4(B), 5TDA(A), 5TSQ(A), 5UEB(A), 5UQZ(A), 5V2O(A), 5VGB(B), 5W2I(A), 5WGI(A), 5WUC(A), 5X9L(A), 5XJ5(A), 5Y0M(A), 5YCE(A), 5YKZ(A), 5YSQ(A), 5Z6D(A), 5ZRY(A), 6A27(A), 6ANZ(A), 6B29(A), 6BD0(A), 6BXD(A), 6CB7(A), 6CPB(B), 6DCM(A), 6DOP(A), 6E4L(A), 6E85(A), 6ELC(A), 6EVN(A), 6F5C(A), 6FFA(A), 6FM5(A), 6FSN(A), 6FZ6(A), 6G96(A), 6GG1(A), 6GQC(A), 6GY5(A), 6H5W(A), 6HER(A), 6HNI(A), 6HX0(A), 6I1A(B), 6IGG(A), 6IWV(B), 6J98(A), 5KY0(B), 5L9A(A), 5LJM(A), 5LS7(D), 5LZK(A), 5M2O(B), 5MBX(A), 5MPW(A), 5N41(A), 5NHU(J), 5NSA(A), 5O15(A), 5O9M(A), 5OL4(C), 5OVO(A), 5R0D(B), 5SZC(A), 5TIF(A), 5TVO(B), 5UEJ(A), 5UUK(B), 5V3N(B), 5VGL(A), 5W4A(C), 5WJP(A), 5WWD(A), 5XA5(B), 5XK6(A), 5Y46(A), 5YDD(A), 5YLG(A), 5YUG(A), 5ZBY(A), 5ZT3(A), 6A2Q(A), 6AO9(A), 6B5K(A), 6BIO(A), 6BXG(A), 6CBR(A), 6CTZ(A), 6DFP(A), 6DQH(A), 6E55(D), 6ECT(A), 6ELM(A), 6EWH(A), 6F5Z(C), 6FG8(B), 6FM7(A), 6FTF(B), 6G1C(V), 6GAJ(A), 6GG7(C), 6GQD(A), 6GZ0(A), 6H8G(A), 6HFM(A), 6HOA(A), 6HXM(A), 6I3B(A), 6IH0(A), 6IX1(B), 6JAL(A), 5KY4(B), 5L9Z(B), 5LJP(A), 5LT5(A), 5LZN(A), 5M2P(A), 5MDU(A), 5MR1(A), 5N4B(C), 5NJ9(B), 5NT7(A), 5O1L(A), 5O9Q(A), 5OL9(A), 5OXZ(B), 5R4Q(A), 5T39(A), 5TJZ(A), 5TW9(A), 5UFY(A), 5UWZ(A), 5V44(A), 5VHG(A), 5W83(A), 5WK0(A), 5X4R(A), 5XAV(A), 5XKX(A), 5Y4T(A), 5YDE(A), 5YMX(B), 5YVK(A), 5ZCY(A), 5ZW7(A), 6A56(B), 6AR0(A), 6B6U(A), 6BM5(A), 6BXR(A), 6CBU(A), 6CWM(A), 6DG4(A), 6DTV(A), 6E5F(A), 6EDV(A), 6ELV(A), 6EWL(A), 6F6M(A), 6FGC(A),

6FMB(A), 6FTO(C), 6G1I(A), 6GBI(A), 6GHT(A), 6GQZ(A), 6GZ8(A), 6H8O(A), 6HFQ(A), 6HPH(A), 6HYO(A), 6I4E(G), 6IIP(A), 6IX2(B), 6JCH(A), 5KY5(B), 5LAL(B), 5LJX(A), 5LUN(A), 5M0W(A), 5M33(A), 5MFA(A), 5MSZ(A), 5N6F(A), 5NJO(A), 5NUE(A), 5O2X(A), 5OBT(E), 5OLL(A), 5P9V(A), 5R8Q(A), 5T3B(A), 5TKW(A), 5U3A(A), 5UJC(A), 5UX1(A), 5V5H(A), 5VJT(A), 5W8O(B), 5WKR(A), 5X57(A), 5XBC(A), 5XLU(B), 5Y4Z(A), 5YDN(A), 5YNX(B), 5YWR(B), 5ZDM(A), 5ZWU(A), 6A5D(B), 6AT4(B), 6B8F(A), 6BO0(A), 6C1X(A), 6CD9(A), 6D0A(A), 6DGA(A), 6DUB(A), 6E5Y(A), 6EF7(A), 6ENI(A), 6EWM(A), 6F8P(A), 6FI2(A), 6FMC(A), 6FU9(B), 6G44(A), 6GCF(A), 6GI4(B), 6GRE(B), 6GZU(A), 6H96(A), 6HG7(A), 6HQC(A), 6HZG(A), 6I5O(D), 6IIY(A), 6IY4(I), 6JEB(A), 6JIW(A), 6JSA(A), 6JZ2(A), 6KIA(B), 6KU0(B), 6L5H(B), 6LUH(B), 6MGC(A), 6N0X(C), 6NHX(A), 6O15(B), 6OJL(A), 6OZD(A), 6P89(A), 6PLH(C), 6PZL(A), 6Q4R(A), 6QDI(A), 6QXR(A), 6R4Z(A), 6RNV(A), 6RY0(A), 6S8K(B), 6SD8(X), 6SLL(B), 6STL(B), 6SYV(A), 6T9Q(A), 6TGU(A), 6TTN(A), 6U2S(A), 6UKF(X), 6UYR(B), 6VK6(C), 6W46(C), 6WN5(A), 6XMI(C), 6XZU(B), 6YFI(A), 6Z9K(A), 7BQP(A), 6JJT(D), 6JTB(A), 6K0P(A), 6KII(A), 6KWZ(A), 6L8S(A), 6M0E(A), 6MIC(A), 6N1B(A), 6NIB(A), 6O19(A), 6OM5(A), 6P28(A), 6P8J(B), 6PLJ(A), 6Q00(B), 6Q4W(B), 6QE0(A), 6R09(A), 6R5J(C), 6RO6(F), 6RY3(A), 6S95(B), 6SE1(A), 6SMT(C), 6SU3(A), 6T02(B), 6TBI(F), 6TIF(B), 6TV2(D), 6U4Z(A), 6ULL(A), 6V1C(A), 6VO5(D), 6W47(B), 6WT8(A), 6XPJ(C), 6Y01(B), 6YII(A), 6ZCO(A), 7BZK(A), 6JK2(A), 6JU1(A), 6K39(A), 6KIS(A), 6KXT(A), 6LAC(A), 6M1V(A), 6MM2(A), 6N4L(A), 6NK0(B), 6O2V(A), 6OPL(A), 6P29(B), 6PBM(A), 6PNV(A), 6Q10(A), 6Q5A(A), 6QHJ(A), 6R1D(B), 6R7V(A), 6RRV(A), 6RYG(A), 6S9K(B), 6SHU(A), 6SOO(A), 6SU5(A), 6T0Y(A), 6TCC(A), 6TJ2(B), 6TVE(P), 6U54(B), 6ULO(A), 6V7G(A), 6VTB(A), 6W4L(A), 6X6Z(A), 6XTJ(A), 6Y4E(A), 6YIP(A), 6ZEG(C), 7C0D(K), 6JK4(A), 6JUF(B), 6K82(B), 6KJK(A), 6KZJ(C), 6LD1(A), 6M76(A), 6MRR(A), 6N6J(A), 6NLQ(C), 6O40(B), 6ORI(A), 6P2C(C), 6PCD(D), 6PQK(D), 6Q1H(G), 6Q5O(A), 6QJA(A), 6R1G(B), 6RB4(A), 6RS4(B), 6RYZ(B), 6SA5(A), 6SID(A), 6SPO(A), 6SUN(A), 6T2T(A), 6TCV(B), 6TJR(A), 6TWP(A), 6U66(A), 6UOF(A), 6VAG(B), 6VVN(B), 6W70(A), 6X7N(A), 6XWE(A), 6Y56(A), 6YJS(B), 6ZPE(A), 7CN7(C), 6JLE(E), 6JV0(A), 6KBX(B), 6KLZ(A), 6L0O(A), 6LG2(B), 6M80(C), 6MU0(A), 6N87(A), 6NNR(B), 6O4M(A), 6OS7(B), 6P2L(A), 6PCZ(A), 6PT8(B), 6Q1M(A), 6Q62(A), 6QP1(A), 6R1M(A), 6RG2(B), 6RVQ(A), 6S07(A), 6SAU(A), 6SIG(D), 6SQP(B), 6SVL(F), 6T3X(A),

6TEQ(B), 6TN1(A), 6TWT(B), 6UAQ(A), 6UQ8(A), 6VG5(B), 6VYD(A), 6W9O(A), 6X8O(A), 6XXJ(A), 6Y5U(A), 6YP6(A), 7A3H(A), 7JID(A), 6JM5(B), 6JV7(A), 6KC5(B), 6KO8(A), 6L0V(E), 6LK1(A), 6M8N(A), 6MX3(A), 6N9H(A), 6NP3(A), 6O5I(A), 6OSX(A), 6P3N(A), 6PFX(B), 6PUQ(A), 6Q2P(B), 6Q6R(G), 6QPK(A), 6R2I(B), 6RI6(A), 6RVU(A), 6S0P(A), 6SBA(B), 6SJ3(A), 6SQX(B), 6SWI(A), 6T6E(A), 6TFR(B), 6TN5(A), 6TYJ(A), 6UBL(B), 6UQV(A), 6VH6(A), 6VZX(B), 6WCV(B), 6XFJ(B), 6XY1(C), 6Y65(I), 6YSK(A), 7A5M(A), 7K3T(A), 6JNY(B), 6JWF(A), 6KFA(A), 6KQS(A), 6L1P(A), 6LL8(A), 6M9M(A), 6MYI(D), 6NDT(B), 6NRH(A), 6O5K(A), 6OVI(C), 6P5H(B), 6PHI(A), 6PVJ(A), 6Q3P(C), 6Q7D(A), 6QSP(B), 6R2W(L), 6RJI(A), 6RW0(A), 6S2M(A), 6SBF(A), 6SJ8(A), 6SSD(A), 6SXT(A), 6T6H(A), 6TGJ(A), 6TRJ(A), 6TYU(A), 6UF3(A), 6USC(B), 6VHU(A), 6W0V(B), 6WEY(A), 6XFU(B), 6XY7(A), 6Y7Q(A), 6YWN(A), 7ADR(F), 7KB2(A), 6JPT(A), 6JWJ(C), 6KFN(A), 6KSR(A), 6L27(A), 6LQF(A), 6MBB(B), 6N0D(B), 6NE2(A), 6NSV(A), 6O6J(A), 6OVM(R), 6P7Z(A), 6PJV(A), 6PYM(A), 6Q41(B), 6Q9L(A), 6QVF(D), 6R33(A), 6RK0(A), 6RWT(A), 6S5W(B), 6SCB(B), 6SJA(B), 6SSH(A), 6SYG(A), 6T7O(A), 6TGK(C), 6TRK(B), 6TZN(A), 6UFE(A), 6UTC(A), 6VIA(A), 6W1G(A), 6WFN(A), 6XIG(A), 6XYA(B), 6YA6(B), 6Z4W(A), 7AF2(A), 7ODC(A), 6JQB(A), 6JWM(A), 6KFS(A), 6KTH(A), 6L4V(A), 6LQN(A), 6MDW(A), 6N0K(A), 6NFR(A), 6NZS(A), 6O8L(A), 6OXJ(B), 6P80(A), 6PKH(A), 6PZD(A), 6Q43(C), 6QAZ(A), 6QW0(B), 6R3M(A), 6RNI(A), 6RXA(A), 6S6C(A), 6SCQ(A), 6SLD(A), 6ST4(A), 6SYJ(B), 6T84(A), 6TGS(A), 6TSM(A), 6TZX(A), 6UID(A), 6UXE(D), 6VJU(A), 6W3D(A), 6WGM(A), 6XIP(C), 6XYB(A), 6YDR(A), 6Z96(A), 7BQI(A), 8ABP(A)

Dataset B : Dataset B is composed of 226 protein structures obtained by processing the database of NMR chemical shifts used for the training of the neural network TALOS-N.[9] For 226 proteins of this database, a structure was determined by NMR. We decided to pick up the first conformers of these NMR structures to build a NMR structure database.

1F7W, 1RZW, 1HZE, 2JZR, 2H25, 1XX3, 2K0G, 2JUH, 2E4J, 1MYF, 1BBN, 1Z2D, 2FCD, 1IJZ, 1YV8, 1N5H, 2DLR, 2BDO, 1XNA, 1LFU, 2HR9, 2K5P, 2DNY, 1XOX, 1POZ, 2JXY, 1Z09, 1ZRP, 1CEJ, 1BNO, 2KID, 1T0V, 1M12, 1TR4, 1M8B, 2IXQ, 1R2A, 1FSP, 2GAQ, 2B1W, 2GJY, 2KOG, 1OVQ, 1VZS, 1BA9, 1S7E, 1JJJ, 1KRS, 2K78, 1XYU, 1MWB, 2CZN, 2D9F, 1SJG, 1D2B, 1F2H,

1PVE, 2H35, 1W4U, 2JTO, 2JPU, 1L8Y, 1OQA, 2JQN, 2AI6, 2H7D, 2HLW, 2KJB, 2JRH, 2AX5, 2JR2, 1URK, 2OFN, 2RTU, 2DO6, 2L84, 1KD6, 1CYE, 2FE9, 1BM6, 2KC3, 1PUT, 2LLN, 1Z2F, 2JRC, 1BYM, 2JWT, 2H8W, 2LLX, 1MVG, 1OTR, 1E3T, 1D1D, 2K9D, 2JNW, 2LD6, 2JNB, 1SOY, 2O4E, 1WLP, 2HM8, 1R8P, 1J8I, 1F43, 1RKN, 2K07, 2AAV, 1FHS, 1B2T, 2AAS, 1YHO, 1I56, 2EVN, 2COM, 2LHA, 2JQ8, 1XUT, 1EZT, 1J5H, 1U3N, 2K18, 2H3F, 1F0Z, 1Y9X, 1E17, 1ON4, 1S79, 2ASY, 1XXE, 1K1C, 1QKF, 2KQK, 1X4C, 3P4K, 1JXS, 1HY8, 1B8Q, 1X32, 1TMW, 1Q59, 1SE7, 2RO5, 1Z00, 2LC6, 1YZS, 1P4S, 2L0X, 1PFL, 1WVZ, 2KH9, 1Z86, 1CFF, 2KMN, 2JT0, 1J0Q, 1M2E, 1RHW, 2FHM, 1YCM, 2JPH, 2P80, 1NZP, 2KX9, 2M3M, 2P3M, 1DF3, 1Q53, 2GO0, 1KRI, 2ERS, 2ARW, 2GZP, 1QQI, 2GT3, 1XFR, 2M86, 3PHY, 2BUG, 1N87, 2LSB, 1WZ0, 1JBH, 1S62, 1JYT, 2FTU, 2RN4, 1SSF, 2HSY, 1LA3, 2LUO, 1V49, 1DGQ, 2UZ5, 1RW5, 2STW, 1TKN, 1IY3, 1NWV, 1Q9P, 2GPF, 1I4V, 1YO4, 1VRE, 1EUB, 2KH2, 1Z7P, 1PM6, 2I85, 1YUU, 1O1W, 2JRL, 2FO8, 2I8N, 1ZDV, 1AZF, 1TBA, 2MKW, 1SLJ, 1M31, 1WH4, 1BKQ, 1AB2, 1AEY, 2DDY, 1CEE, 1Q6A

# References


(1) Kabsch, W.; Sander, C. Dictionary of protein secondary structure: pattern recognition of hydrogen-bonded and geometrical features. *Biopolymers* **1983**, *22*, 2577–2637.

(2) DeLano, W. The PyMOL Molecular Graphics System, Version 1.2r3pre. 2002.

(3) Wang, G.; Dunbrack, R. PISCES: a protein sequence culling server. *Bioinformatics* **2003**, *19*, 1589–1591.

(4) Jones, D. T. Protein secondary structure prediction based on position-specific scoring matrices. *J Mol Biol* **1999**, *292*, 195–202.

(5) Frishman, D.; Argos, P. Knowledge-based protein secondary structure assignment. *Proteins* **1995**, *23*, 566–579.

(6) Si, D.; Moritz, S. A.; Pfab, J.; Hou, J.; Cao, R.; Wang, L.; Wu, T.; Cheng, J. Deep Learning to Predict Protein Backbone Structure from High-Resolution Cryo-EM Density Maps. *Sci Rep* **2020**, *10*, 4282.

(7) Pfab, J.; Phan, N.; Si, D. DeepTracer for fast de novo cryo-EM protein structure modeling and special studies on CoV-related complexes. *Proc Natl Acad Sci U S A* **2021**, *118*, e2017525118.

(8) Berman, H.; Bhat, T.; Bourne, P.; Feng, Z.; Gilliland, G.; Weissig, H.; Westbrook, J. The Protein Data Bank and the challenge of structural genomics. *Nat Struct Biol* **2000**, *7 Suppl*, 957–959.

(9) Shen, Y.; Bax, A. Protein structural information derived from NMR chemical shift with the neural network program TALOS-N. *Methods Mol Biol* **2015**, *1260*, 17–32.
